# Comparative genomic and antimicrobial resistance profiles of *Salmonella* strains isolated from pork and human sources in Sichuan, China

**DOI:** 10.3389/fmicb.2025.1515576

**Published:** 2025-03-03

**Authors:** Haojiang Zuo, Yang Yang, Minchuan Su, Weifeng Huang, Jian Wang, Gaopeng Lei, Ximei Kong, Peng Chen, Yun Leng, Qiwu Yuan, Yuanyuan Zhao, Yanfang Miao, Ming Li, Xin Xu, Shihui Lu, Hui Yang, Lvbo Tian

**Affiliations:** ^1^West China School of Public Health and West China Fourth Hospital, Sichuan University, Chengdu, China; ^2^West China-PUMC C.C. Chen Institute of Health, Sichuan University, Chengdu, China; ^3^Food Safety Monitoring and Risk Assessment Key Laboratory of Sichuan Province, Chengdu, China; ^4^Chengdu Centre for Disease Control and Prevention, Chengdu, China; ^5^Sichuan Provincial Centre for Disease Control and Prevention, Chengdu, China; ^6^Chenghua Centre for Disease Control and Prevention, Chengdu, China; ^7^College of Pharmacy, Youjiang Medical University for Nationalities, Baise, China; ^8^West China School of Stomatology, Sichuan University, Chengdu, China; ^9^Sichuan Entry-Exit Inspection and Quarantine Bureau, Chengdu, China

**Keywords:** *Salmonella*, antimicrobial resistance, whole genome sequencing, human, pork

## Abstract

**Introduction:**

*Salmonella* detection in retail pork is increasing, yet studies on its antimicrobial resistance (AMR) profiles and genomic characteristics remain limited. Moreover, it is still unclear whether certain *Salmonella* sequence types (STs) are consistently or rarely associated with pork as a transmission source. Sichuan province, the largest pork-production region in China, provides a critical setting to investigate these dynamics.

**Methods:**

In this study, 213 *Salmonella* strains isolated from pork and human sources (2019–2021) underwent phenotypic AMR testing and whole-genome sequencing (WGS).

**Results:**

Resistance profiling revealed a higher prevalence of AMR in the pork-derived strains, particularly in veterinary-associated antibiotics. We identified STs not observed in pork in this study, such as ST23 (*S*. Oranienburg) and the poultry-commonly associated ST32 (*S*. Infantis), suggesting potential non-pork transmission routes for these *Salmonella* STs. To quantify sequence type diversity within each sample source, we introduced the sequencing type index (ST index = number of different STs/ total isolates). The ST index was 32% (49/153) for human-derived isolates and 20% (12/60) for pork-derived isolates. PERMANOVA analysis revealed significant differences in the structural composition of sequence types between human- and pork-derived isolates (*p* = 0.001), indicating that pork may harbor specific *Salmonella* STs more frequently.

**Discussion:**

These findings highlight the role of pork as a reservoir for certain *Salmonella* STs, while also implying potential non-pork transmission pathways. The ST index represents a novel metric for assessing *Salmonella* diversity across different sample sources, offering a better understanding of genetic variation and transmission dynamics.

## 1 Introduction

*Salmonella* spp. are major pathogens responsible for a substantial burden of foodborne diseases worldwide (Fu et al., 2022; Fay et al., 2023). It is estimated that between 200 million and 1 billion instances of *Salmonella* infection occur worldwide annually, culminating in approximately 93 million cases of gastroenteritis and 155,000 deaths (Chlebicz and Slizewska, 2018; Castro-Vargas et al., 2020; Hung et al., 2017). In China, specifically in the Sichuan Province, salmonellosis accounts for approximately 9.87 million cases of gastroenteritis annually, making it a critical region for research on foodborne illnesses caused by *Salmonella* (Zuo et al., 2022; Su et al., 2023; Kong et al., 2022).

While poultry-associated *Salmonella* has attracted widespread attention (Gu et al., 2023), *Salmonella* detection in retail pork is increasing, with industrialized pork production contributing to the global dissemination of *Salmonella enterica* (Li et al., 2024; Li et al., 2022). Sichuan, the largest pork producer in China, exceeded 60 million pigs annually in 2021 and 2022, and is projected to produce 70 million by 2027, with over 70% from large-scale farms (The_People's_Government_of_Sichuan_Province, 2023). Sichuan’s large-scale pork production may uniquely affect local *Salmonella* resistance and genetic profiles. Meanwhile, Sichuan’s multi-ethnic population, diverse dietary habits, and varied geography contribute to the complexity of *Salmonella* transmission through food, water, and other environmental routes (Elias, 2024; Sun et al., 2023; Shu et al., 2024; Shu et al., 2023).

Previous research in Sichuan primarily investigated the prevalence and phenotypic resistance of *Salmonella* isolates from pig and other sources over a decade ago (Li et al., 2013). However, to our knowledge, no study has comprehensively compared *Salmonella* strains from both human and pork sources in this region. Furthermore, detailed data on the resistance patterns and genomic profiles of *Salmonella* spp. in Sichuan pork production chain are scarce (Guan et al., 2022). This highlights the need for a comprehensive analysis to better understand *Salmonella* transmission dynamics and its potential public health implications in this key pork production hub.

Among the over 2,600 serotypes of *Salmonella enterica*, *S.* Typhimurium, and *S*. Derby are frequently associated with human infections linked to pork consumption (De Sousa et al., 2024; Chu et al., 2024). However, it remains unclear whether certain *Salmonella* sequence types (STs) are consistently or rarely associated with pork as a transmission source. Additionally, the genomic diversity and distribution of *Salmonella* STs in Sichuan remain poorly understood.

By employing antimicrobial susceptibility testing and whole-genome sequencing (WGS), this study investigated the antimicrobial resistance (AMR) profiles, genomic diversity, and sequence type distribution of *Salmonella* strains isolated from pork and human sources in Sichuan, the largest pork production hub in China. Meanwhile, this study also introduces the sequencing type index (ST index), a simple and effective metric for quantifying *Salmonella* diversity across different sample sources. These findings aim to support the development of targeted prevention and control strategies that integrate veterinary and public health initiatives.

## 2 Materials and methods

### 2.1 *Salmonella* isolation and serotyping

**Figure 1:**
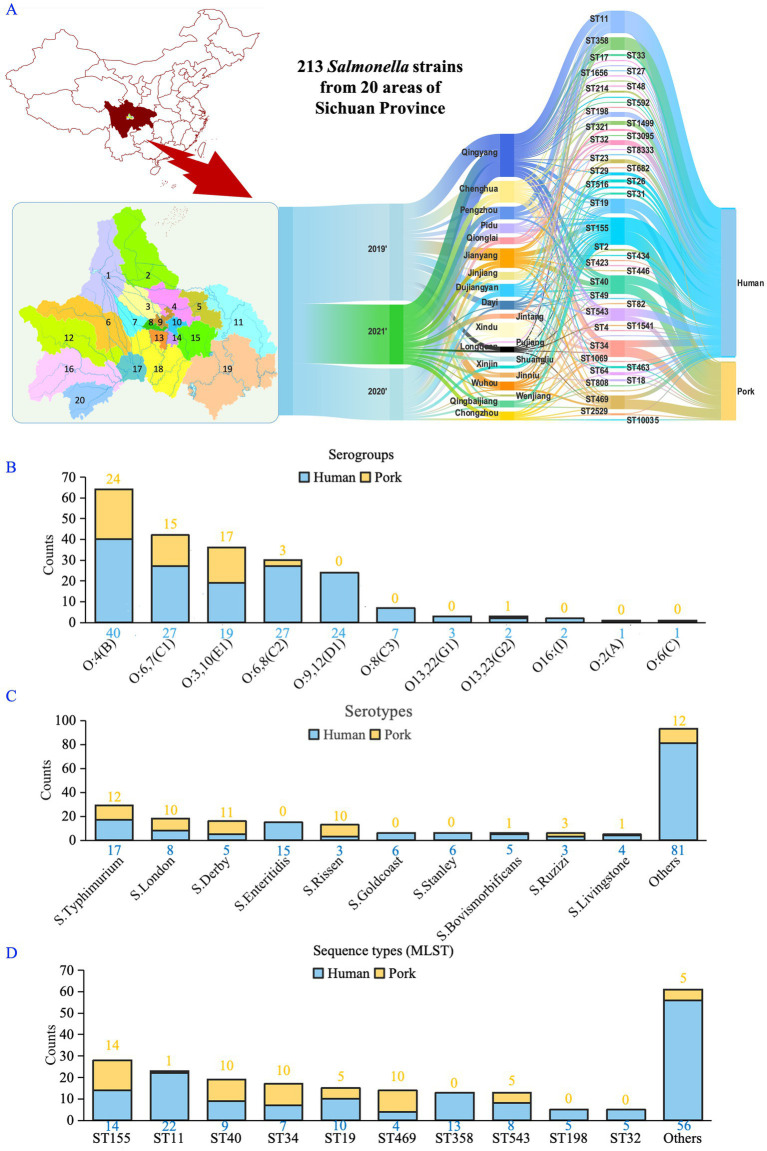
Classification and distribution of *Salmonella* isolates from human and pork sources. **(A)** Spatiotemporal distribution of *Salmonella* isolates (human-derived, *n* = 153; pork-derived, *n* = 60). 1: Dujiangyan; 2: Pengzhou; 3: Pidu; 4: Xindu; 5: Qingbaijiang; 6: Chongzhou; 7: Wenjiang; 8: Qingyang; 9: Jinniu; 10: Chenghua; 11: Jintang; 12: Dayi; 13: Wuhou; 14: Jinjiang; 15: Longquanyi; 16: Qionglai; 17: Xinjin; 18: Shuangliu; 19: Jianyang; 20: Pujiang. **(B)** Serogroup classification of isolates based on the slide agglutination test, showing the presence of 11 serogroups. **(C)** The dominant serotypes identified in both human and pork-derived isolates, with *Salmonella* Typhimurium and *S*. London being the most prevalent. **(D)** Primary sequence types (STs) identified through MLST analysis. *S*. London (ST155) and *S.* Typhimurium (ST11, ST19, ST34) were the most common and found in both sources.

The study, conducted from 2019 to 2021, involved collecting pork samples from 20 regions in the Sichuan Province to isolate *Salmonella*. Simultaneously, human-derived *Salmonella* were isolated from 28 medical institutions across 20 regions in the Sichuan Province, including the five main urban districts of the capital city: Wuhou, Jinjiang, Chenghua, Qingyang, and Jinniu District; as well as 15 surrounding areas: Dayi, Pujiang, and Jintang County; Qionglai, Dujiangyan, Pengzhou, and Jianyang City; Shuangliu, Longquanyi, Pidu, Qingbaijiang, Xindu, Xinjin, Chongzhou and Wenjiang District (Figure 1A).

In adherence to the Foodborne Disease Surveillance Program’s confidentiality protocols, details regarding the sample size and sampling plan are restricted-internal information (Zhou et al., 2024). Consequently, this study does not present the detection rate or other related findings. However, the 20 regions included in this study account for nearly one-quarter of Sichuan’s total population and approximately one-third of the province’s gross domestic product (GDP), ensuring a broadly representative selection of pork sources and medical institutions in this region.

Pork samples were collected from various retail sources in Sichuan Province. Sampling sites included traditional Chinese wet markets, where pork was stored at ambient temperature and directly exposed to the environment, and supermarkets, where pork was kept under refrigerated conditions. To ensure representativeness, samples were randomly selected from different retail locations, with each sample weighing no less than 200 g. The samples were labeled, individually packed in sterile sampling bags, and promptly placed in insulated boxes with ice packs for immediate transport to the laboratory.

In the laboratory, Pork samples were tested for *Salmonella* in accordance with the National Food Safety Standards of China (GB 4789.4–2016). Approximately 25 g of each sample was processed in a biosafety cabinet, pre-enriched in Buffered Peptone Water (BPW) at 36°C for 8–18 h, followed by selective enrichment in Tetrathionate Broth (TTB) at 42°C for 18–24 h. The enriched cultures were streaked onto CHROMagar *Salmonella* plates, Hektoen Enteric (HE) agar, and/or Xylose Lysine Deoxycholate (XLD) agar, then incubated at 36°C for 18–24 h. The suspected strains were then identified using a BioMérieux VITEK 2 compact system. For serotyping, single colonies derived from Swarm agar after overnight incubation at 37°C were analyzed to detect O-antigens and H-antigens using slide agglutination tests. These tests were performed using *Salmonella* antiserum kits (60 V) sourced from the Statens Serum Institute in Copenhagen, Denmark and Beijing Land Bridge Technology in Beijing, China (Kong et al., 2022; Zhou et al., 2024; GB4789.4-2016, 2016).

For human samples, approximately 2 g of freshly voided fecal samples were collected in clean, dry, wide-mouth containers and transported to the laboratory within 1 h using insulated transport boxes. The fecal samples were directly inoculated onto CHROMagar *Salmonella* plates, *Salmonella*- XLD agar and/or *Shigella* (SS) agar. Suspected colonies were subjected to biochemical and serological identification as described above.

### 2.2 Antimicrobial susceptibility testing

Drug resistance in *Salmonella* isolates was evaluated using the microbroth dilution kit provided by Xingbai, Shanghai, China (Kong et al., 2022). This involved a drug susceptibility test employing the microbroth dilution technique to determine the resistance of *Salmonella* strains to a panel of eight antibiotics: ampicillin (AMP, ≥32 μg/mL), ampicillin/sulbactam (AMS, ≥32/16 μg/mL), tetracycline (TET, ≥16 μg/mL), chloramphenicol (CHL, ≥32 μg/mL), trimethoprim/sulfamethoxazole (SXT, ≥4/76 μg/mL), ciprofloxacin (CIP, ≥1 μg/mL), ceftazidime (CAZ, ≥16 μg/mL), and cefotaxime (CTX, ≥4 μg/mL).

Resuscitated *Salmonella* strains were streaked onto TSA (Tryptic Soy Agar) plates and incubated at 36°C for 18–24 h. Isolated colonies were then suspended in sterile saline, homogenized, and adjusted to 1.5 × 10^8^ CFU/mL using a McFarland densitometer.

A 60 μL aliquot of the bacterial suspension was diluted in 12 mL of nutrient broth, thoroughly mixed, and then transferred into a sterile multi-channel pipette reagent reservoir for reagent dispensing. Sterile nutrient broth was used as the negative control. Plates were incubated at 35°C for 18–20 h.

Minimum inhibitory concentrations (MICs) were determined using the Sensititre™ Vizion™ Digital MIC Viewing System (TREK Diagnostic Systems, Thermo Fisher Scientific) according to Clinical and Laboratory Standards Institute (CLSI) guidelines. Quality control (QC) strains, including *Escherichia coli* ATCC25922, *Staphylococcus aureus* ATCC29213, *Klebsiella pneumoniae* ATCC 700603, *Enterococcus faecalis* ATCC 29212 and *Pseudomonas aeruginosa* ATCC27853, were cultured under the same conditions to ensure test accuracy. QC strain MIC values were confirmed to fall within CLSI-defined reference ranges. Strains were categorized as multi-drug resistant (MDR) if they exhibited resistance to three or more classes of antibiotics (Parzygnat et al., 2024).

### 2.3 Whole-genome sequencing and bioinformatics analysis

*Salmonella* strains were streaked onto LB solid medium and cultured at 37°C for 12 h. A single colony was then inoculated into 200 mL of LB liquid medium and cultured at 37°C for approximately 12 h at 150 rpm. The cell biomass was harvested by centrifugation at 12,000 × g for 10 min. Genomic DNA was extracted using the Magbeads FastDNA Kit for Soil (MP Biomedicals, United Kingdom) according to the manufacturer’s protocol. Purified genomic DNA was quantified, and high-quality DNA was used for library construction. DNA quality was evaluated using a 1% agarose gel, and DNA concentration was measured using the Qubit dsDNA High-Sensitivity Assay Kit (Thermo Fisher Scientific, Waltham, MA, United States).

The draft genome sequencing of *Salmonella* strains was performed on the Illumina NovaSeq 6,000 platform. Genomic DNA was sheared into ~400 bp fragments using a Covaris M220 Focused Acoustic Shearer, following the manufacturer’s instructions. Sequencing libraries were prepared using the NEXTFLEX Rapid DNA-Seq Kit (Revvity, Waltham, MA, USA). The preparation steps included end-repair and phosphorylation of the 5′ ends, A-tailing of the 3′ ends, and ligation of sequencing adapters. Adapter-ligated products were enriched by PCR, and the resulting libraries were used for paired-end 2 × 150 bp sequencing on the Illumina NovaSeq 6,000 platform (Illumina Inc., San Diego, CA, USA). The sequencing quality metrics for all samples demonstrated high reliability, with an average Q30 score of 92.7%, which exceeds the minimum acceptable score of 80%, an average N50 length of 255,215.9 base pairs, and an average scaffold number of 138.4. The average coverage was 91.9% based on K-mer analysis and 100.0% based on reads mapping (NGS). Detailed individual data are provided in the “QC” sheet of the Excel file provided as Supplementary file 1.

Raw data were processed using Fastp (version 0.19.6) and *de novo* assembly was performed using SOPAdenovo2 (Chen et al., 2018; Luo et al., 2012). Antibiotic resistance and virulence genes were identified using the Comprehensive Antibiotic Resistance Database (CARD) and the Virulence Factor Database (VFDB; Akter et al., 2023). Diamond was employed as the alignment tool with an E-value threshold of ≤1e-5 (Hu et al., 2023). Analyses were performed on assembled reads to ensure accurate annotation. The thresholds for identity and coverage to include antimicrobial resistance and virulence genes were set at no less than 80 and 60%, respectively (Worley et al., 2018; Liu et al., 2024). Core virulence genes were defined as those found in at least 98% of the analyzed *Salmonella* isolates, reflecting their essential roles in the pathogenicity of *Salmonella enterica*.

Multi-locus sequence typing (MLST) was conducted using BioNumerics version 7.6 (Zhou et al., 2024). Phylogenetic trees were constructed using core genome MLST (cgMLST) on the BigsDB platform with 3,002 loci (Alikhan et al., 2018). Multiple sequence alignments were performed using MAFFT, and the tree was built using FastTree with the parameters-nt-gtr. Visualization of the tree was conducted on the TVBot and iTOL websites. This cgMLST-based phylogeny provides a high-resolution comparison of genetic relationships among *Salmonella* isolates. To assess the robustness of the results, we also performed the analysis using the scheme developed by the INNUENDO consortium for *Salmonella enterica*, which consists of 3,255 loci (Silva et al., 2018; Llarena et al., 2018; Fredriksson-Ahomaa et al., 2024).

We have deposited all 213 genomes in the NCBI database. All sequence data can be accessed via the following link: NCBI PRJNA1082412 and PRJNA1081960.

### 2.4 Statistical analyses

Descriptive statistics were used to summarize the data, with categorical variables reported as numbers and percentages (%) and continuous variables, when included, reported as means and standard deviations (SD). For group comparisons, the chi-square test was used for categorical variables when expected frequencies were sufficient, while Fisher’s exact test was applied when sample sizes were small. For continuous variables, the t-test was used for normally distributed data, and the rank-sum test was applied for non-normally distributed data. Spearman correlation analysis was conducted to assess relationships between phenotypic AMR and the presence of resistance and virulence genes. A *p*-value of ≤0.05 was considered statistically significant for all tests. Statistical significance was assessed using R version 4.1.2 (R Foundation for Statistical Computing, Vienna, Austria; Zhou et al., 2024; Conway et al., 2017; Zhou et al., 2022; Noble et al., 2025).

## 3 Results

### 3.1 Distribution of *Salmonella* isolates

From 2019 to 2021, we obtained a total of 213 *Salmonella* isolates from human (*n* = 153) and pork (*n* = 60) sources. The slide agglutination test identified 11 serogroups among these strains (Figure 1B), with *S*. Typhimurium and *S*. London being the dominant serotypes (Figure 1C). Among the identified STs derived from MLST, *S*. London (ST155) and *S.* Typhimurium (ST11, ST19, and ST34) were the most prevalent, appearing in both human and pork sources (Figure 1D).

Notably, about one-third of the serotypes identified using the slide agglutination differed from STs derived from MLST (Supplementary file 1). Given the high reproducibility of sequencing-based methods and the study’s focus on *Salmonella*’s AMR profiles and genomic characteristics, subsequent analyses were conducted using STs instead of serotypes. We have also conducted sensitivity analyses in the *Salmonella* isolates (*n* = 124) with consistent serotypes and STs to assess the robustness of the main findings, particularly focusing on antimicrobial resistance profiles and evolutionary tree analysis.

### 3.2 Phenotypic antimicrobial resistance

**Figure 2:**
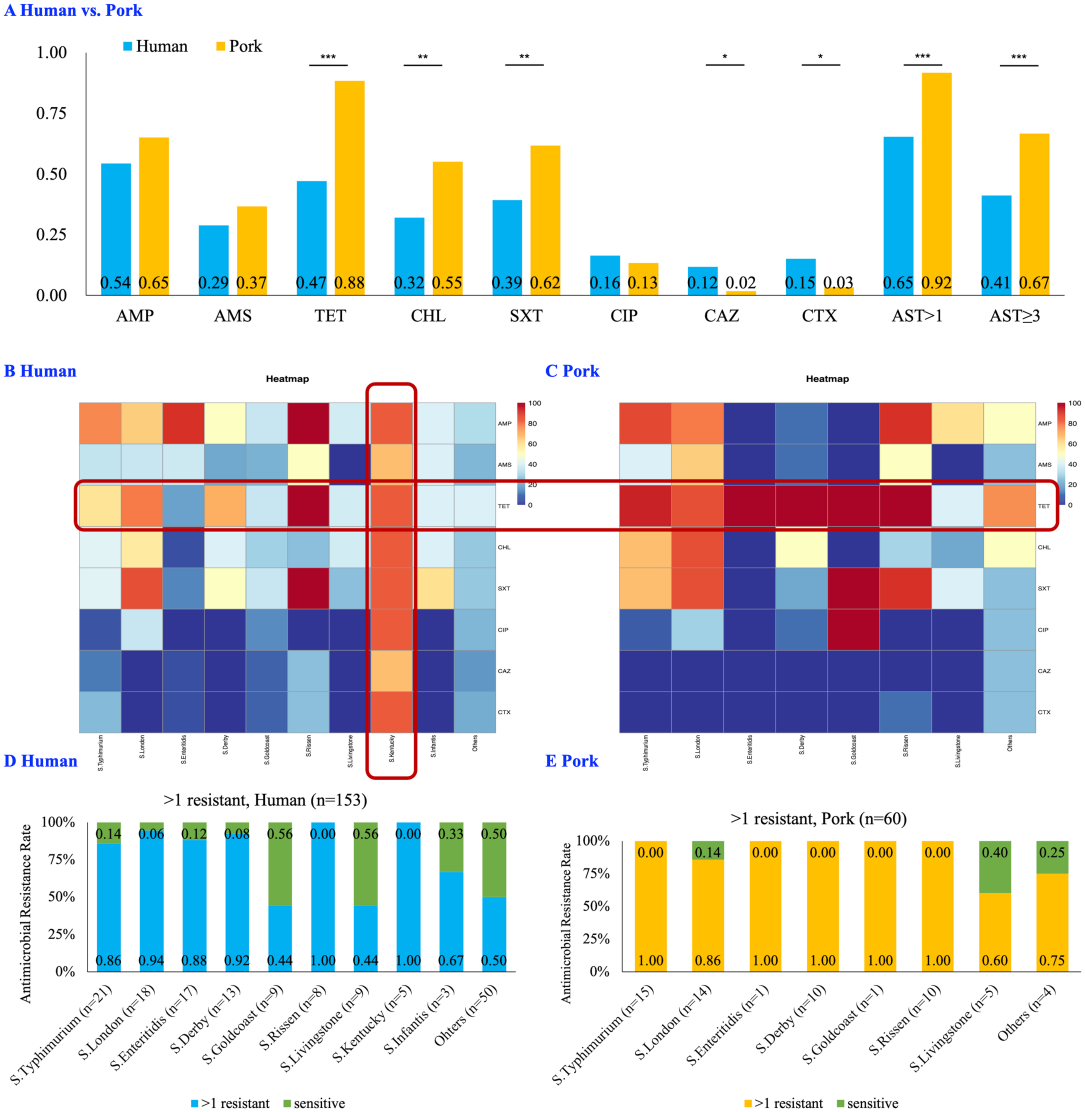
Comparative analysis of antimicrobial resistance between Human- and Pork-derived *Salmonella* isolates (*n* = 213). **(A)** Bar chart comparing the resistance profiles of human-derived (*n* = 153) and pork-derived (*n* = 60) isolates. The proportion of TET, CHL, SXT, AST > 1 and AST ≥ 3 was significantly higher in pork-derived isolates compared to human-derived isolates (*p* < 0.01). The chi-square test was used for categorical variables when expected frequencies were sufficient, while Fisher’s exact test was applied when sample sizes were small. **(B,C)** Heatmaps showing the resistance profiles of the major STs in human-derived **(B)** and pork-derived **(C)** isolates. *S*. Kentucky (ST198), identified exclusively in human samples, exhibited the highest resistance levels. The scale (0–100) indicates the percentage of resistance. **(D,E)** Proportions of resistant strains (AST > 1) across the most prevalent STs in human-derived **(D)** and pork-derived **(E)** isolates. No significant differences were observed in resistance prevalence between these STs. STs: sequence types, AMP: ampicillin, AMS: ampicillin/sulbactam, TET: tetracycline, CHL: chloramphenicol, SXT: trimethoprim/sulfamethoxazole, CIP: ciprofloxacin, CAZ: ceftazidime, CTX: cefotaxime; AST > 1: strains resistant to more than one antibiotic; AST ≥ 3/MDR: multidrug-resistant strains, strains resistant to more than one antibiotic; *p < 0.05; **p ≤ 0.01; ***p ≤ 0.001.

Analysis of the AMR patterns revealed significant differences among the isolates. Chi-square test/Fisher’s exact test showed that pork-derived isolates exhibited higher rates of resistance to TET (88.3% vs. 47.1%, *p* < 0.001), CHL (55.0% vs. 32.0%, *p* = 0.003), and SXT (61.7% vs. 39.2%, *p* = 0.003) compared to those of human-derived isolates. Conversely, the human isolates demonstrated significantly higher resistance to CAZ (11.8% vs. 1.7%, *p* = 0.031) and CTX (15.0% vs. 3.3%, *p* = 0.016) compared to that of the pork-derived isolates. There was no significant difference in resistance to AMP, AMS, CIP, and CAZ between the human-and pork-derived isolates (Figure 2A).

Regarding broader antimicrobial susceptibility, the proportion of strains resistant to more than one antibiotic (Antimicrobial Susceptibility Testing, AST > 1) was significantly higher in the pork-derived isolates than that in the human-derived isolates (91.7% vs. 65.4%, *p* < 0.001). A similar trend was observed for isolates resistant to three or more antibiotics (AST ≥ 3 / MDR, 66.7% vs. 41.2%, *p* = 0.001, Figure 2A). When considering all tested antibiotics, the average number of resistant antibiotics per strain was significantly higher in pork-derived isolates (3.25 per strain) than in human-derived isolates (2.44 per strain; *p* = 0.005). Similar results were also observed in the 124 *Salmonella* isolates with consistent serotypes and STs (Supplementary Figure S1).

Among the major *Salmonella* STs, the *S.* Kentucky (ST198), which was exclusively found in human samples, exhibited the highest levels of resistance (Figure 2B). Moreover, TET resistance was significantly higher in pork-derived isolates compared with those from humans (Figures 2B,C). However, the antimicrobial resistance prevalences between the most prevalent *Salmonella* STs isolated from pork samples and humans did not significantly differ (Figures 2D,E), suggesting similar resistance profiles between the two groups. Combined with the finding that *S*. London (ST155) and *S.* Typhimurium (ST11, ST19, and ST34) were present in both human and pork sources, these results indicated that pork may serve as a reservoir for certain *Salmonella* strains.

### 3.3 Genotypic AMR and correlation analyses with phenotypes

Comparative analysis of the CARD gene profiles in the *Salmonella* strains isolated from humans and pork sources revealed significant differences in the prevalence of various AMR genes. A total of 128 resistance genes were identified in human-derived *Salmonella* strains, and 92 in pork-derived strains. Notably, according to the Chi-square test/Fisher’s exact test, genes such as *qacL*, *aadA*, *sul3*, *aadA2*, *tet(A)*, *dfrA12*, *tetM*, *cmlA1*, *sul2*, and *floR* exhibited significantly higher frequencies in pork-derived isolates. For instance, *qacL*, which encodes a quaternary ammonium compound resistance protein associated with resistance to disinfectants and antiseptics, was detected 43.3% of pork-derived isolates compared to only 14.4% of human-derived isolates (*p* < 0.001). Similarly, *tet(A)*, a gene that confers tetracycline resistance by encoding an efflux pump protein, showed a significantly higher prevalence in pork-derived isolates compared to human-derived isolates (71.7% vs. 40.5%, *p* < 0.001, Supplementary Table S1, Supplementary file 1).

**Figure 3:**
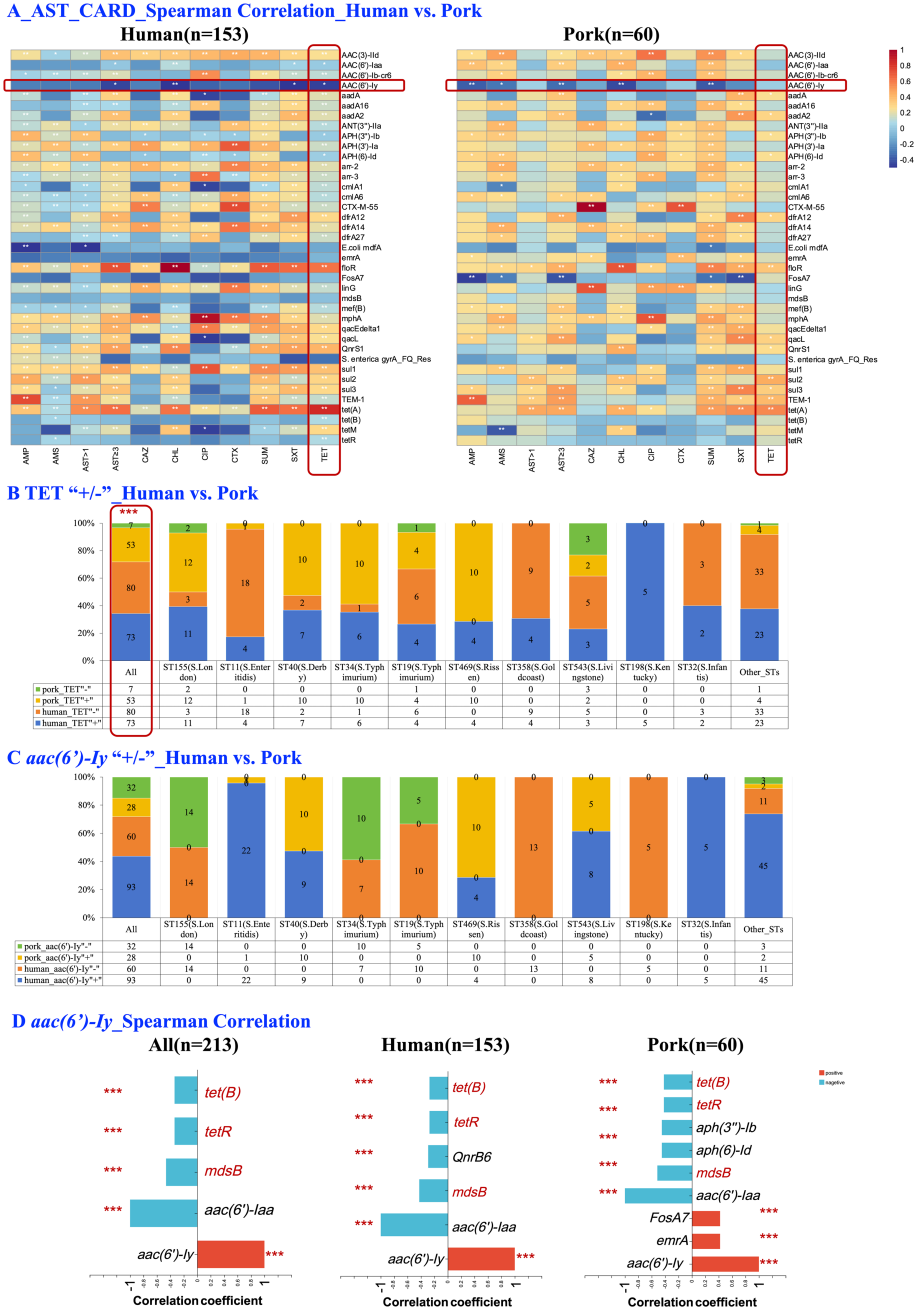
Comparative analyses of phenotypic and genotypic antimicrobial resistance in human- and pork-derived *Salmonella* isolates (*n* = 213). **(A)** Spearman correlation analysis between phenotypic antimicrobial resistance (measured by susceptibility testing) and the presence of resistance genes in human-derived isolates (**A**, *n* = 153) and pork-derived isolates (**B**, *n* = 60). Significant correlations were observed between TET resistance and resistance genes such as *tet(A)*, *sul2*, *bla*_TEM-1_ and *aac(6′)-Iy*. **(B)** Comparison of TET resistance prevalence (+/−) between human and pork-derived isolates. Pork-derived isolates exhibit a significantly higher proportion of TET resistance compared to human-derived isolates (88.3% vs. 47.7%, *p* < 0.001). **(C)** Comparison of the presence (+/−) of the *aac(6′)-Iy* gene between human- and pork-derived isolates. No clear preference for the presence of the gene within the same sequence types was observed. **(D)** Spearman correlation analysis of the *aac(6′)-Iy* gene with other resistance genes. Significant negative correlations with tetracycline resistance genes, such as *tet(B)* and *tetR*, were observed across all isolates (*n* = 213, *p* < 0.001), with similar trends seen in both human-derived (*n* = 153, *p* < 0.001) and pork-derived isolates (*n* = 60, *p* < 0.001). Spearman correlation analysis was applied for this figure. The chi-square test was used for categorical variables when expected frequencies were sufficient, while Fisher’s exact test was applied when sample sizes were small. Scale: 0–1, representing correlation coefficient (*ρ*); TET, tetracycline; *p < 0.05; **p ≤ 0.01; ***p ≤ 0.001.

Figure 3A shows the Spearman correlation analysis results between phenotypic AMR (measured by susceptibility testing) and the presence of resistance genes (genotypic antimicrobial resistance). Significant positive correlations were observed between TET resistance and the presence of *tet(A)*, *sul2*, and *bla*_TEM-1_ in both human- and pork-derived isolates. Interestingly, the gene *aac(6′)-Iy*, which encoded aminoglycoside acetyltransferase that typically does not confer resistance to aminoglycosides under normal conditions (The_Comprehensive_Antibiotic_Resistance_Database, 2023), exhibited a significant negative correlation with TET resistance (*p* < 0.05), a phenomenon only observed in human-derived *Salmonella* isolates.

To further investigate this relationship, we analyzed the co-occurrence patterns of *aac(6′)-Iy* and TET resistance among human-derived isolates. Of the 153 human strains, 22.9% (35/153) carried only TET resistance, 35.9% (55/153) carried only the *aac(6′)-Iy* gene, 24.8% (38/153) carried both, and 16.3% (25/153) carried neither. A Chi-square test revealed a statistically significant difference in the distribution patterns of *aac(6′)-Iy* gene and TET resistance (*p* = 0.035). In contrast, pork-derived isolates exhibited a significantly higher proportion of TET resistance compared to human-derived isolates (88.3% vs. 47.7%, *p* < 0.001; Figure 3B). However, within the same sequence types, no clear trend was observed (Figures 3B,C). Finally, in the Spearman correlation analysis between *aac(6′)-Iy* and resistance genes across all isolates (*n* = 213), significant negative correlations were observed with specific tetracycline resistance genes, such as *tet(B)* and *tetR* (*p* < 0.001; Figure 3D). These significant negative correlations were consistently observed in human-derived isolates (*n* = 153, *p* < 0.001; Figure 3D), pork-derived isolates (*n* = 60, *p* < 0.001; Figure 3D) and 124 *Salmonella* isolates with consistent serotypes and STs (Supplementary Figure S2).

### 3.4 Virulence gene profiling

Our examination of the VFDB showed a prevalence of 98–100% for core virulence genes, such as *galF*, *stiH*, *stiC*, *spaS*, *spaR*, *sipD*, and *sicA* in both sources, underscoring their strong conservation and essential roles in the survival of the pathogen. These include genes involved in lipopolysaccharide biosynthesis (*galF*) and components of the type III secretion system (*spaS*, *spaR*, *sipD*, and *sicA*) that facilitate the injection of effector proteins into host cells, promoting invasion, immune evasion, and bacterial survival within host cells (Kelly et al., 2024; Miletic et al., 2021).

Significant variations in the prevalence of several virulence genes between human- and pork-derived isolates were observed, indicating potential differences in virulence power. Notably, according to the Chi-square test/Fisher’s exact test, the positivity rates of *sciJ*, *csgD*, and *sciS*/*icmF*-like were significantly different between the two sources. For example, *sciJ*, a potentially novel gene, was detected in 68.0% of human-derived isolates and 98.3% of pork-derived isolates (*p* < 0.001). Conversely, *csgD*, a key regulator involved in biofilm formation and curli production (Gonzalez et al., 2024), showed a prevalence of 70.6% in human-derived isolates and 26.7% in pork-derived isolates (*p* < 0.001). Such differences may suggest distinct adaptations in virulence strategies depending on the host environment. Similarly, several other virulence factors, such as *sciO* (98.3% vs. 75.8%, *p* < 0.05), *sciP* (98.3% vs. 74.5%, *p* < 0.05), and *sciL* (98.3% vs. 74.5%, *p* < 0.05, Supplementary Table S2), also had higher prevalence in pork-derived isolates than those in human samples, with each displaying near-universal prevalence in pork-derived isolates compared with a lower prevalence in human samples.

Furthermore, certain genes showed strikingly low prevalence in isolates from both sources, indicating that they either had a less important role in the overall virulence or a specialized function under specific environmental conditions (Supplementary Table S2).

### 3.5 Phylogenetic analyses

**Figure 4:**
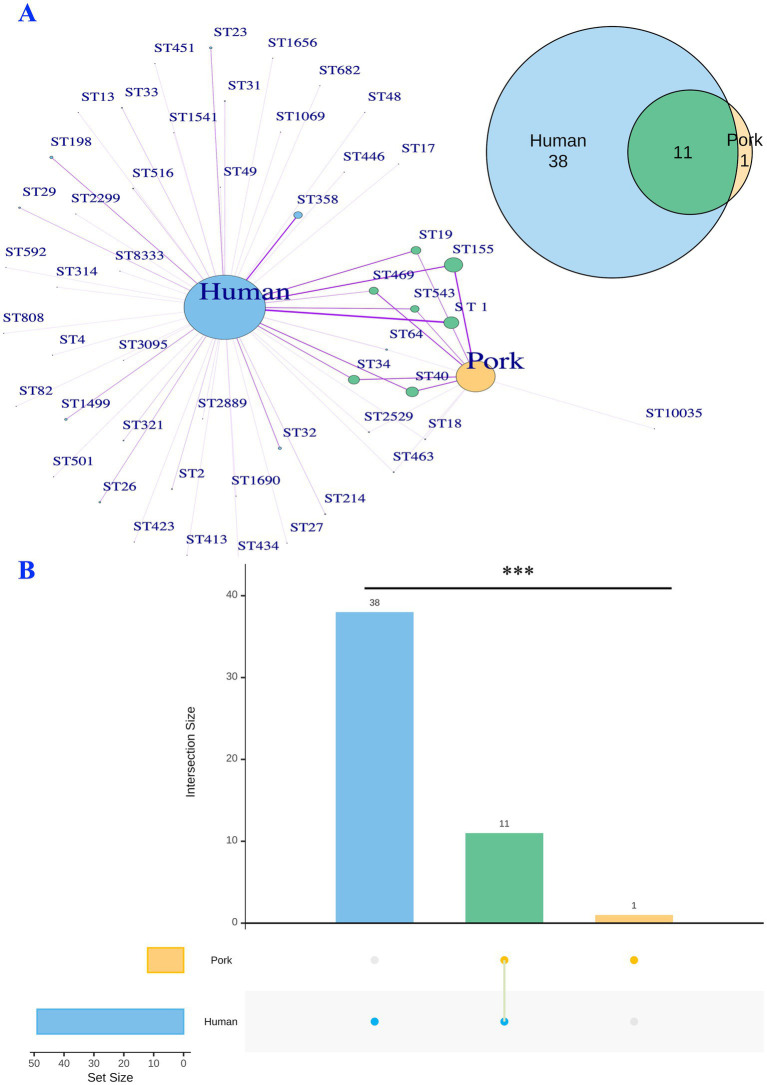
Comparative analysis of STs between human- and pork-derived *Salmonella* isolates (*n* = 213). **(A)** A network diagram and Venn diagram illustrating the relationships of shared and unique STs between human-derived (*n* = 153) and pork-derived (*n* = 60) isolates. A total of 11 STs were found in both human and pork sources, with ST11 (*S. enteritidis*), ST155 (*S*. London), ST19 (*S.* Typhimurium), ST40 (*S*. Derby), ST543 (*S*. Livingstone), and ST469 (*S*. Rissen) being the predominant sequencing types. **(B)** An UpSet plot showing the distribution of STs across human- and pork-derived isolates. Human-derived isolates exhibited a higher ST index (32%, 49/153) compared to pork-derived isolates (20%, 12/60), indicating greater sequence type diversity in human samples. The structural composition of STs was significantly different between the two groups (PERMANOVA analysis, *p* = 0.001). STs: sequence types; ST index = number of different STs/sample size; *p < 0.05; ** *p* ≤ 0.01; *** *p* ≤ 0.001.

**Figure 5:**
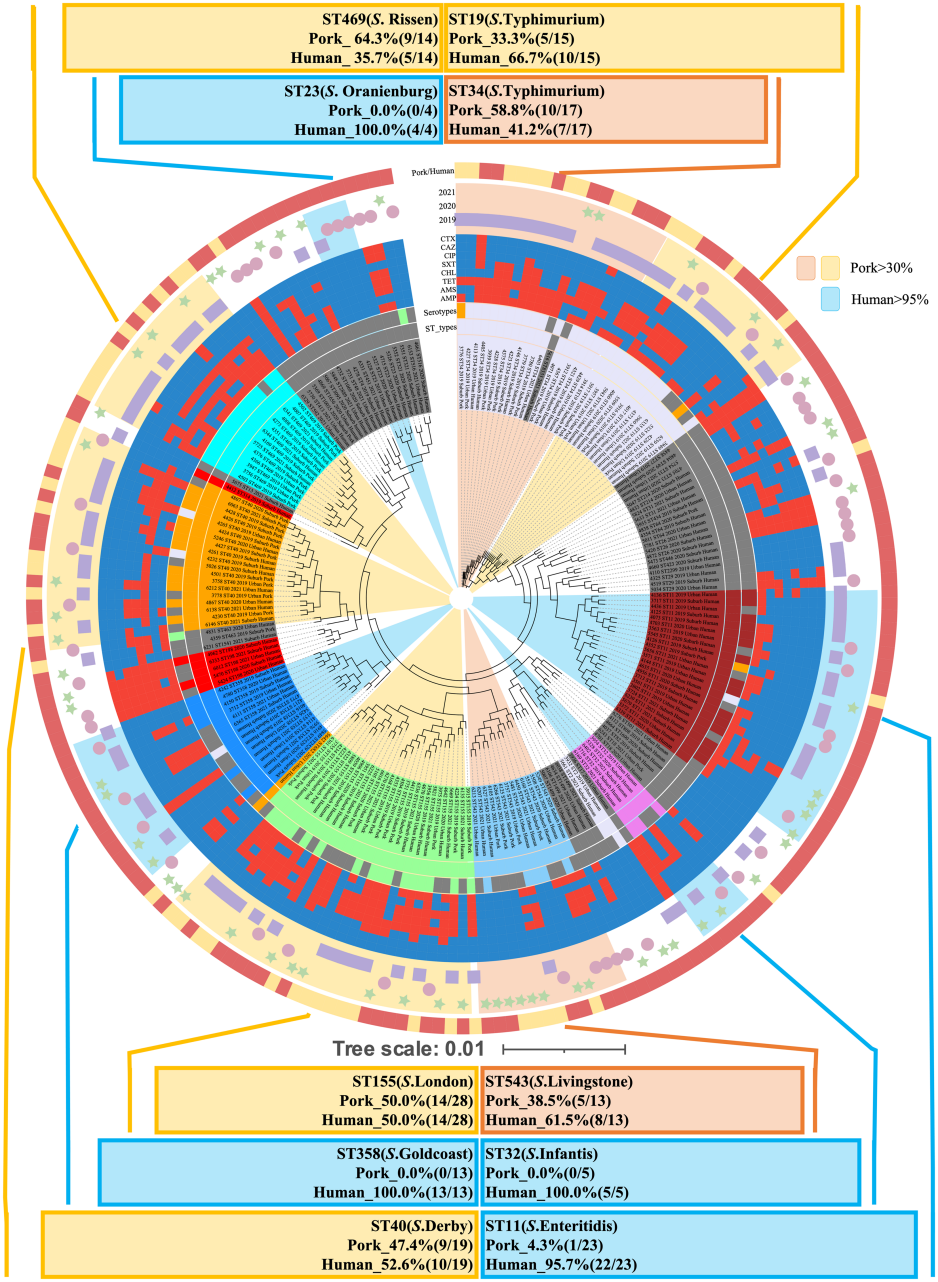
The cgMLST-based phylogenetic tree of 213 *Salmonella* isolates using BigsDB. The tree illustrates the genetic relationships among human-derived (*n* = 153) and pork-derived (*n* = 60) *Salmonella* isolates. A total of 50 sequence types (STs) were identified through in silico MLST analysis. Predominant STs, including ST11 (*S. enteritidis*), ST155 (*S*. London), ST19 (*S.* Typhimurium), ST40 (*S*. Derby), ST543 (*S*. Livingstone), and ST469 (*S*. Rissen), were shared between human and pork sources, indicating their potential role in cross-species transmission. In contrast, ST23 (*S*. Oranienburg), ST32 (*S.* Infantis), ST198 (*S*. Kentucky), ST358 (*S*. Goldcoast), and ST1499 (*S.* Bovismorbificans) were exclusively detected in human-derived isolates in this study. A branch length of 0.01 indicates 1% allelic variation.

As illustrated in Figure 4A, the 213 *Salmonella* isolates were classified into 50 STs through *in silico* MLST analysis. Among these, 11 STs were common in isolates from both human and pork sources. The predominant STs included: ST11 (*S. enteritidis*, *n* = 23), ST155 (*S*. London, *n* = 28), ST19 (*S.* Typhimurium, *n* = 15), ST40 (*S*. Derby, *n* = 19), ST543 (*S*. Livingstone, *n* = 13), and ST469 (*S.* Rissen, *n* = 14). These STs were identified in both human and pork sources, highlighting their potential role in cross-species transmission (Figures 4A, 5). In contrast, ST23 (*S.* Oranienburg, *n* = 4), ST32 (*S.* Infantis, *n* = 5), ST198 (*S.* Kentucky, *n* = 5), ST358 (*S.* Goldcoast, *n* = 13), and ST1499 (*S.* Bovismorbificans, *n* = 4), etc., were only detected in isolates from human sources (Figures 4B, 5).

In Figure 4B, an UpSet plot was generated to visualize the shared and unique STs between human- and pork-derived *Salmonella* isolates. There are 12 STs in pork-derived isolates and 49 STs in human-derived isolates. Among them, 38 STs were unique to human-derived isolates, 1 ST was unique to pork-derived isolates, and 11 STs were shared between both sources. To assess the diversity of sequence types within each source, we developed the ST index, defined as the ratio of the number of different STs to the total number of isolates from a given source (ST index = number of different STs/total isolates). This index provides a simple measure of ST diversity within each sample source. The ST index for human-derived *Salmonella* isolates was 32% (49/153), whereas that for pork-derived *Salmonella* isolates was 20% (12/60). The structural composition of STs was significantly different between human- and pork-derived *Salmonella* strains, as confirmed by PERMANOVA analysis using the Jaccard dissimilarity distance (*p* = 0.001; Figure 4B; Noble et al., 2025), indicating that pork may harbor certain *Salmonella* sequence types more frequently, whereas human isolates display a broader range of STs (Figure 5). Similar results were also observed in the analysis using the cgMLST scheme developed by the INNUENDO consortium for *Salmonella enterica* (Supplementary Figures S3, S4).

## 4 Discussion

*Salmonella enterica* spp. are major pathogens responsible for foodborne diseases worldwide, with the World Health Organization classifying MDR *Salmonella* as high-priority pathogens due to their increasing prevalence (Bolkenov et al., 2024; Farha et al., 2023; Giraud et al., 2024). The advent of WGS has significantly enhanced our understanding of the population structure, drug resistance gene transmission, and antimicrobial resistance epidemiology of *Salmonella*. *enterica* (Doublet et al., 2024; Kiss et al., 2019). Notably, WGS also enables the identification of *Salmonella* STs, which are critical for understanding genetic diversity and transmission dynamics. However, it remains unclear whether the distribution of *Salmonella* STs in pork differs from that in humans. The modernization of agriculture and international commerce have contributed to the widespread distribution of *Salmonella* from pig-related sources (Li et al., 2024; Chu et al., 2024). Sichuan Province, as the foremost region for pork production in China, plays a critical role in understanding the transmission dynamics of *Salmonella*. In this study, we conducted a pilot evaluation of the AMR and genomic characteristics of *Salmonella* strains isolated from pork and human sources in Sichuan Province (2019–2021), contributing to a better understanding of the regional epidemiology and potential public health risks associated with this largest pork production hub in China.

### 4.1 AMR

Our findings highlight the significant differences in AMR between *Salmonella* strains isolated from humans and pork sources, with more pronounced resistance in pork-derived isolates. Notably, the antimicrobial resistance prevalences to TET, CHL, and SXT were higher in pork-derived strains than those in human-derived strains, which is an alarming trend. These antibiotics are commonly used in veterinary practice and these findings suggest that their routine use in animal husbandry may contribute to elevated resistance levels (Sheng et al., 2023).

In particular, the significant TET resistance observed in the pork-derived isolates (88.3%) reflects its widespread use as a growth promoter in livestock, leading to antibiotic-resistant bacterial populations. This has been a persistent issue in the veterinary and public health sectors (Saady et al., 2024). As a result, the resistance rates observed in our study were similar to those in eastern China (Li et al., 2022) but significantly higher than in countries that implemented strict regulations earlier, such as Europe (Roasto et al., 2023) and Brazil (Rodrigues et al., 2020; Rabello et al., 2020). For instance, TET resistance in Brazil was reported to be 20.3% in swine isolates and 17% in human isolates (Rodrigues et al., 2020), whereas our study revealed markedly higher levels of 88.3% in pork-derived isolates and 47.1% in human-derived isolates. This disparity likely stems from the delayed prohibition of antibiotic use as growth promoters in China (Rabello et al., 2020; Wen et al., 2022; Zhao et al., 2023).

Although China officially prohibited the use of antibiotics as growth promoters in animal husbandry in July 2020 as part of a national strategy to combat antimicrobial resistance (Wen et al., 2022), data from 2018 to 2020 indicate that antimicrobials continue to be widely used in veterinary medicine, particularly tetracyclines and macrolides (Zhao et al., 2023), which may contribute to the high resistance observed in pork-derived *Salmonella* isolates in this study. These comparisons underscore the urgent need for effective monitoring and enforcement of regulations on antibiotic use in veterinary practice to prevent the increase in antimicrobial-resistant bacteria that can be transmitted to humans through the food chain.

Conversely, antimicrobial resistance prevalences to CAZ and CTX were significantly higher in human-derived *Salmonella* isolates compared with that of those derived from pork, which may be mainly attributed to antibiotic use in human healthcare. CAZ and CTX are third-generation cephalosporins that are widely used in human medicine, particularly for treating serious bacterial infections. Resistance to these antibiotics is less common in pork because third-generation cephalosporins are generally reserved for critical cases in veterinary medicine and companion animals (Pepin-Puget et al., 2020).

In summary, the elevated resistance to TET, CHL, and SXT in pork isolates highlights the influence of veterinary antibiotic use, while the increased resistance to CAZ and CTX in human isolates reflects their application in clinical medicine. These findings emphasize the complexity of AMR transmission pathways. Leveraging tools such as machine learning may offer promising approaches for analyzing AMR patterns and guiding preventive interventions (Tang et al., 2023; Farhat et al., 2023).

### 4.2 Gene analyses

We found 128 AMR genes in human-derived *Salmonella* and 94 in pork-derived strains. In comparison, Central China reported 82 AMR genes in *Salmonella* from pork and other animals combined (Gu et al., 2023). This suggests higher AMR gene prevalence in Sichuan pork, likely due to its concentrated, industrialized production, increasing selective pressure for AMR.

The significant prevalence of resistance genes, such as *qacL* and *tet(A),* which confer resistance to quaternary ammonium compounds and TET, in pork-derived isolates reflects the selective pressures from their historical use as growth promoters and prophylactics in pig farming (Wen et al., 2022; Zhao et al., 2023; Sielski Galvao Soares et al., 2023). The disparity in TET resistance between pork- and human-derived *Salmonella* isolates may reflect the different antibiotic use practices in veterinary and human medicine, indicating more frequent or prolonged exposure to TET in veterinary medicine. Significant correlations were observed between TET and *tet(A)* and *sul2* in both human- and pork-derived isolates. However, the mechanisms underlying these associations, including the potential role of horizontal gene transfer, remain unclear and require further investigation to identify the drivers of resistance gene dissemination (Oladeinde et al., 2021).

Interestingly, we observed a significant negative correlation between the *aac(6′)-Iy* gene, an aminoglycoside N-acetyltransferase gene, and TET resistance in human-derived *Salmonella* strains. A similar negative correlation was also observed in the overall population (*n* = 213), as well as in both pork-derived (*n* = 60) and human-derived (*n* = 153) *Salmonella* strains, between *aac(6′)-Iy* and specific tetracycline resistance genes, such as *tet(B)* and *tetR*. Two explanations may account for this negative observation: source diversity and potential competition between resistance genes. From the perspective of source diversity, different sample sources might harbor different resistance genotypes. For example, pork-derived strains, frequently exposed to tetracycline antibiotics in pig farming, are enriched for TET resistance genes (Li et al., 2022). In contrast, strains from other sources, such as poultry, beef, and the environment, preferentially carry *aac(6′)-Iy* (Shu et al., 2023; Guan et al., 2022; Emond-Rheault et al., 2020; Aleri et al., 2022; Mao et al., 2021). Since human infections often result from multiple sources, this mixed effect could statistically explain why *aac(6′)-Iy* and TET resistance appear negatively correlated in human strains.

Alternatively, fitness burden and metabolic competition may explain the negative correlation (Christaki et al., 2020). The *aac(6′)-Iy* gene, which is cryptic and widely present in *Salmonella* species, does not confer aminoglycoside resistance under normal conditions. Instead, it has been implicated in intracellular survival within *Salmonella*-containing vacuoles and plays a role in host infection (The_Comprehensive_Antibiotic_Resistance_Database, 2023; Krath, 2022). On the other hand, tetracycline resistance typically involves energy-intensive mechanisms, such as efflux pumps, ribosomal protection, or enzymatic inactivation (Wisniewski et al., 2024; Pavelquesi et al., 2021). Co-expression of *aac(6′)-Iy* and TET resistance genes might impose significant fitness costs on bacterial cells, limiting their simultaneous presence due to resource competition.

### 4.3 Phylogenetic analyses

Our analysis identified several predominant *Salmonella* STs shared between human and pork sources, including ST155 (*S*. London), ST11 (*S. enteritidis*), ST40 (*S*. Derby), ST34 (*S.* Typhimurium), ST19 (*S.* Typhimurium), ST469 (*S*. Rissen), and ST543 (*S*. Livingstone; Figures 1D, 4, 5). This overlap in sequence types suggests that pork is likely a major source of *Salmonella* infections in humans.

Our phylogenetic findings align with previous studies showing that *S*. Derby (ST40, *n* = 19) was common in isolates from both human and pork sources (Chu et al., 2024; Yuan et al., 2022). While, in our study, ST23 (*S.* Oranienburg), ST32 (*S.* Infantis), ST198 (*S.* Kentucky), ST358 (*S.* Goldcoast), and ST1499 (*S.* Bovismorbificans) were found exclusively in human samples. These sequence types are commonly associated with poultry, cattle, and other environmental sources (Jiang et al., 2023). Our findings implied that the distribution of *Salmonella* sequencing types in pork may differ from those observed in humans.

### 4.4 ST index

We developed the concept of the ST index as a simple and effective measure of sequence type diversity within a sample source (ST index = number of different STs/total isolates). This concept does not apply to individual isolates because each colony corresponds to only one sequence type (Zuo et al., 2020; Xie et al., 2023). It is particularly useful when applied to larger datasets, providing an overview of genetic diversity.

By comparing the ST index across different sample sources, we can assess their relative contribution to transmission dynamics. For instance, a higher ST index in *Salmonella* isolates from a sample source indicates greater genetic diversity, suggesting that the source serves as a reservoir for a broader variety of *Salmonella* strains, and may contribute to a more diverse range of STs being transmitted to humans. In contrast, a lower ST index may reflect a more restricted reservoir, with the ability to transmit only a limited number of certain *Salmonella* STs to humans.

In our findings, the ST index for human-derived *Salmonella* isolates was 32%, significantly higher than the 20% observed for pork-derived isolates (*p* < 0.001). This suggests a complex epidemiological landscape for human infections, likely influenced by multiple reservoirs, including pork, alongside poultry, cattle, dairy products, environmental sources, etc. (Selim et al., 2022; Roobab et al., 2023; Mukherjee et al., 2019). In contrast, the lower ST index for pork-derived isolates implies a transmission dynamic dominated by certain types, such as ST40 (*S*. Derby), which is commonly linked with pork (De Sousa et al., 2024; Chu et al., 2024).

By quantifying genetic diversity, the ST index helps assess the potential contribution of specific reservoirs to *Salmonella* infections, supporting targeted surveillance and control measures. These findings, centered on sequence type analysis, emphasize the need for expanded investigations into the transmission dynamics and potential reservoirs in the region (Mastroeni et al., 2009). Future studies could incorporate serovar-specific analyses to further elucidate strain-source relationships and *Salmonella* transmission pathways. Additionally, the ST index could also be a useful tool for comparing *Salmonella* STs across different regions.

### 4.5 STs and traditional serotyping

Conventional phenotypic serotyping is a multi-day, labor-intensive process requiring the isolation of single bacterial colonies, preparation of bacterial suspensions, and manual interpretation of agglutination results. This workflow heavily relies on skilled laboratory technicians and is, therefore, time-consuming and resource-intensive. To address these challenges, there is an increasing need for adaptable approaches that can rapidly and accurately discriminate different *Salmonella* strains without depending solely on traditional serotyping (Tran et al., 2024).

In contrast, sequence type-based approaches offer several advantages and are increasingly employed in *Salmonella* research, as they provide a standardized, highly reproducible, and widely accepted framework for investigating *Salmonella* epidemiology (Yan et al., 2021; Uelze et al., 2020). While sequence typing is inherently predictive and has its own limitations, it provides valuable complementary insights to overcome the challenges of traditional serotyping. Sequencing-based methods streamline the typing process, offering faster, more consistent, and standardized comparisons across isolates. Given these advantages, we adopted STs for subsequent analyses, ensuring a robust and uniform framework for evaluating *Salmonella* strain diversity.

In our study, both traditional serotyping and sequencing typing via MLST were conducted. Notably, discrepancies were observed in approximately one-third of the serotypes when comparing results from the slide agglutination test with MLST predictions. Similar inconsistencies have also been reported in studies conducted by other provincial CDCs (Zhou et al., 2024). These inconsistencies may arise from limitations in serotyping kits, variability in manual interpretation, errors during traditional serotyping such as incomplete serotyping and human misjudgment, and other methodological factors inherent to traditional phenotypic assay.

Nevertheless, it is important to acknowledge that sequencing-based typing, while efficient and highly standardized, is inherently predictive and does not replace the direct detection of antigens through traditional slide agglutination assays. The accuracy of sequence typing can be influenced by sequencing errors, incomplete databases, or mutations that impact gene prediction. Conversely, traditional serotyping, though labor-intensive and subject to variability in manual interpretation, remains a reliable method for detecting antigenic structures. Therefore, both approaches have unique strengths and limitations, and combining traditional and genomic methods may provide a more comprehensive and accurate framework for *Salmonella* typing, particularly in surveillance and outbreak investigations.

### 4.6 Limitation

This study has certain limitations. First, confidentiality protocols under the Foodborne Disease Surveillance Program restricted the release of specific details about the sample size, which may limit the representativeness of the results and hinder regional comparisons. However, the selected regions represent approximately one-quarter of Sichuan’s population and one-third of its GDP (Zhou et al., 2022), which ensures that the human and pork samples are broadly representative despite these limitations.

Second, this study employed a draft genome approach without generating complete genomes, which limited the depth of genomic analysis and may have resulted in inadequate coverage of certain genes. Future studies should consider incorporating PCR to validate specific gene differences (Renuka et al., 2004) and integrating second- and third-generation sequencing technologies to generate complete genomes, enabling more insights into *Salmonella*’s genetic features and resistance mechanisms.

Third, the use of the ST index to compare human-derived and pork-derived samples has inherent limitations. Human-derived samples often encompass diverse sources, including multiple animal and environmental origins, whereas pork-derived samples represent a more singular source. This complexity may obscure the true contribution of each source to the observed diversity in human samples. Therefore, direct comparisons between these two groups using the ST index should be interpreted with caution.

While the ST index remains a useful metric for assessing strain diversity, future research could refine its application by focusing on single-source comparisons, such as pork versus chicken or cattle. Moreover, incorporating serovar-specific ST indexes may provide more meaningful and specific comparisons between isolates, enhancing the interpretation of diversity and transmission dynamics. Finally, as this study serves as a pilot investigation, future studies with larger sample sizes are necessary to validate these findings and further strengthen their robustness.

## 5 Conclusion

This pilot study elucidated the AMR profiles and ST distribution of *Salmonella* strains isolated from human and pork sources in Sichuan Province, the largest pork production hub in China. Pork-derived strains showed higher resistance to veterinary antibiotics (e.g., tetracycline), while human-derived strains exhibited greater resistance to antibiotics used in human healthcare (e.g., cephalosporins). Our phylogenetic analysis revealed overlapping sequence types between human- and pork-derived isolates, suggesting pork may serve as a reservoir for certain *Salmonella* strains. We also identified STs detected only in human samples in this study, such as ST23 (*S*. Oranienburg) and ST32 (*S.* Infantis), suggesting potential non-pork transmission routes for these *Salmonella* STs. This study also introduced the ST index as a simple and effective tool for quantifying the diversity of *Salmonella* sequence types across different sample sources. Collectively, this study advances our understanding of *Salmonella* resistance profiles, genetic diversity, and transmission dynamics in this critical food production hub, emphasizing the urgent need for targeted surveillance and interventions to mitigate associated public health risks.

## Data Availability

The datasets presented in this study can be found in online repositories. The names of the repository/repositories and accession number(s) can be found in the article/Supplementary material. The 213 genomes data can be accessed via the following two accession numbers: PRJNA1082412 and PRJNA1081960.
