# Supplementary material for: Comparative genomic and antimicrobial resistance profiles of Salmonella strains isolated from pork and human sources in Sichuan, China
Source: Front Microbiol. 2025 Mar 3;16:1515576. doi: 10.3389/fmicb.2025.1515576 (PMC11911478; doi:10.3389/fmicb.2025.1515576)
Supplement: Supplementary file 1 [file Data_Sheet_1.docx]

**Supplementary tables and figures**

- [**Table S1 Comparative analysis of AMR between Human and Pork (n=213)** 2](#_Toc190465222)
- [**Table S2 Comparative analysis of virulence genes between Human and Pork (n=213)** 7](#_Toc190465223)
- [**Figure S1 Comparative analysis of antimicrobial resistance between Human- and Pork-derived *Salmonella* isolates (n=124)**. 21](#_Toc190465224)
- [**Figure S2 Spearman correlation analysis of the *aac(6')-Iy* gene with other resistance genes**. 21](#_Toc190465225)
- [**Figure S3 The cgMLST-based phylogenetic tree of 213 *Salmonella* isolates based on the scheme developed by the INNUENDO consortium.** 23](#_Toc190465226)
- [**Figure S4 The cgMLST-based phylogenetic tree of 124 *Salmonella* isolates based on the scheme developed by the INNUENDO consortium.** 25](#_Toc190465227)

**Table S1 Comparative analysis of AMR between Human and Pork (n=213)**

| No | CARD |  | Human(%(95%CI)) (n=153) | Pork(%(95%CI)) (n=60) |
| --- | --- | --- | --- | --- |
| 1 | *tet(A)* | * | 40.5%(33.1–48.4) | 70.0%(57.5–80.1) |
| 2 | *sul2* | * | 37.9%(30.6–45.8) | 60.0%(47.4–71.4) |
| 3 | *floR* | * | 30.7%(24.0–38.4) | 50.0%(37.7–62.3) |
| 4 | *sul3* | * | 19.0%(13.5–25.9) | 45.0%(33.1–57.5) |
| 5 | *dfrA12* | * | 19.0%(13.5–25.9) | 40.0%(28.6–52.6) |
| 6 | *qacL* | * | 14.4%(9.7–20.8) | 43.3%(31.6–55.9) |
| 7 | *aadA2* | * | 15.7%(10.8–22.3) | 40.0%(28.6–52.6) |
| 8 | *aadA* | * | 11.8%(7.6–17.8) | 33.3%(22.7–45.9) |
| 9 | *tetM* | * | 13.1%(8.6–19.3) | 26.7%(17.1–39.0) |
| 10 | *cmlA1* | * | 11.1%(7.1–17.1) | 23.3%(14.4–35.4) |
| 11 | *mdsB* | * | 10.5%(6.5–16.3) | 23.3%(14.4–35.4) |
| 12 | *Salmonella enterica gyrA conferring resistance to fluoroquinolones* | * | 17.6%(12.4–24.5) | 1.7%(0.3–8.9) |
| 13 | *emrA* | * | 7.8%(4.5–13.2) | 18.3%(10.6–29.9) |
| 14 | *FosA7* | * | 7.2%(4.1–12.4) | 18.3%(10.6–29.9) |
| 15 | *tetR* | * | 4.6%(2.2–9.1) | 16.7%(9.3–28.0) |
| 16 | *tet(B)* | * | 4.6%(2.2–9.1) | 16.7%(9.3–28.0) |
| 17 | *aadA15* | * | 0.7%(0.1–3.6) | 6.7%(2.6–15.9) |
| 18 | *aadA8* | * | 0.7%(0.1–3.6) | 6.7%(2.6–15.9) |
| 19 | *TEM-1* |  | 41.2%(33.7–49.1) | 55.0%(42.5–66.9) |
| 20 | *QnrS1* |  | 26.1%(19.8–33.6) | 35.0%(24.2–47.6) |
| 21 | *H-NS* |  | 100.0%(97.6–100.0) | 100.0%(94.0–100.0) |
| 22 | *CRP* |  | 100.0%(97.6–100.0) | 100.0%(94.0–100.0) |
| 23 | *Klebsiella pneumoniae KpnF* |  | 100.0%(97.6–100.0) | 100.0%(94.0–100.0) |
| 24 | *emrR* |  | 100.0%(97.6–100.0) | 100.0%(94.0–100.0) |
| 25 | *cpxA* |  | 100.0%(97.6–100.0) | 100.0%(94.0–100.0) |
| 26 | *emrB* |  | 100.0%(97.6–100.0) | 100.0%(94.0–100.0) |
| 27 | *rsmA* |  | 100.0%(97.6–100.0) | 100.0%(94.0–100.0) |
| 28 | *E.coli ampH beta-lactamase* |  | 100.0%(97.6–100.0) | 100.0%(94.0–100.0) |
| 29 | *baeR* |  | 100.0%(97.6–100.0) | 100.0%(94.0–100.0) |
| 30 | *E.coli acrA* |  | 100.0%(97.6–100.0) | 100.0%(94.0–100.0) |
| 31 | *PmrF* |  | 100.0%(97.6–100.0) | 100.0%(94.0–100.0) |
| 32 | *msbA* |  | 100.0%(97.6–100.0) | 100.0%(94.0–100.0) |
| 33 | *kdpE* |  | 100.0%(97.6–100.0) | 100.0%(94.0–100.0) |
| 34 | *MdtK* |  | 100.0%(97.6–100.0) | 100.0%(94.0–100.0) |
| 35 | *sdiA* |  | 100.0%(97.6–100.0) | 100.0%(94.0–100.0) |
| 36 | *bacA* |  | 100.0%(97.6–100.0) | 100.0%(94.0–100.0) |
| 37 | *E.coli EF-Tu mutants conferring resistance to Pulvomycin* |  | 100.0%(97.6–100.0) | 100.0%(94.0–100.0) |
| 38 | *mdsC* |  | 99.3%(96.4–99.9) | 100.0%(94.0–100.0) |
| 39 | *E.coli soxS with mutation conferring antibiotic resistance* |  | 99.3%(96.4–99.9) | 100.0%(94.0–100.0) |
| 40 | *E.coli soxR with mutation conferring antibiotic resistance* |  | 99.3%(96.4–99.9) | 100.0%(94.0–100.0) |
| 41 | *E.coli UhpT with mutation conferring resistance to fosfomycin* |  | 99.3%(96.4–99.9) | 100.0%(94.0–100.0) |
| 42 | *E.coli GlpT with mutation conferring resistance to fosfomycin* |  | 98.7%(95.4–99.6) | 100.0%(94.0–100.0) |
| 43 | *marA* |  | 98.7%(95.4–99.6) | 98.3%(91.1–99.7) |
| 44 | *E.coli marR mutant conferring antibiotic resistance* |  | 98.7%(95.4–99.6) | 98.3%(91.1–99.7) |
| 45 | *golS* |  | 98.0%(94.4–99.3) | 100.0%(94.0–100.0) |
| 46 | *mdsA* |  | 98.0%(94.4–99.3) | 100.0%(94.0–100.0) |
| 47 | *acrB* |  | 98.0%(94.4–99.3) | 98.3%(91.1–99.7) |
| 48 | *E.coli mdfA* |  | 70.6%(62.9–77.2) | 73.3%(61.0–82.9) |
| 49 | *aac(6')-Iy* |  | 60.8%(52.9–68.2) | 46.7%(34.6–59.1) |
| 50 | *aac(6')-Iaa* |  | 39.2%(31.8–47.1) | 53.3%(40.9–65.4) |
| 51 | *aph(6)-Id* |  | 28.1%(21.6–35.7) | 33.3%(22.7–45.9) |
| 52 | *aph(3'')-Ib* |  | 27.5%(21.0–35.0) | 33.3%(22.7–45.9) |
| 53 | *qacEdelta1* |  | 24.2%(18.1–31.5) | 30.0%(19.9–42.5) |
| 54 | *sul1* |  | 22.2%(16.4–29.4) | 30.0%(19.9–42.5) |
| 55 | *mphA* |  | 15.7%(10.8–22.3) | 13.3%(6.9–24.2) |
| 56 | *arr-3* |  | 12.4%(8.1–18.6) | 13.3%(6.9–24.2) |
| 57 | *aac(3)-IId* |  | 12.4%(8.1–18.6) | 11.7%(5.8–22.2) |
| 58 | *mef(B)* |  | 9.2%(5.5–14.8) | 18.3%(10.6–29.9) |
| 59 | *aac(6')-Ib-cr6* |  | 11.1%(7.1–17.1) | 13.3%(6.9–24.2) |
| 60 | *dfrA14* |  | 10.5%(6.5–16.3) | 10.0%(4.7–20.1) |
| 61 | *aph(3')-Ia* |  | 10.5%(6.5–16.3) | 10.0%(4.7–20.1) |
| 62 | *arr-2* |  | 9.8%(6.0–15.5) | 10.0%(4.7–20.1) |
| 63 | *cmlA6* |  | 7.2%(4.1–12.4) | 15.0%(8.1–26.1) |
| 64 | *aadA16* |  | 8.5%(5.0–14.0) | 10.0%(4.7–20.1) |
| 65 | *dfrA27* |  | 8.5%(5.0–14.0) | 10.0%(4.7–20.1) |
| 66 | *ANT(3'')-IIa* |  | 7.2%(4.1–12.4) | 8.3%(3.6–18.1) |
| 67 | *CTX-M-55* |  | 8.5%(5.0–14.0) | 1.7%(0.3–8.9) |
| 68 | *linG* |  | 7.8%(4.5–13.2) | 3.3%(0.9–11.4) |
| 69 | *QnrB6* |  | 5.2%(2.7–10.0) | 6.7%(2.6–15.9) |
| 70 | *cmlA5* |  | 3.9%(1.8–8.3) | 6.7%(2.6–15.9) |
| 71 | *OXA-10* |  | 3.9%(1.8–8.3) | 6.7%(2.6–15.9) |
| 72 | *catB3* |  | 3.9%(1.8–8.3) | 3.3%(0.9–11.4) |
| 73 | *oqxA* |  | 2.6%(1.0–6.5) | 6.7%(2.6–15.9) |
| 74 | *aadA8b* |  | 3.9%(1.8–8.3) | 3.3%(0.9–11.4) |
| 75 | *OXA-1* |  | 3.9%(1.8–8.3) | 3.3%(0.9–11.4) |
| 76 | *aadA17* |  | 3.3%(1.4–7.4) | 5.0%(1.7–13.7) |
| 77 | *aadA5* |  | 3.9%(1.8–8.3) | 1.7%(0.3–8.9) |
| 78 | *dfrA17* |  | 3.9%(1.8–8.3) | 1.7%(0.3–8.9) |
| 79 | *oqxB* |  | 2.0%(0.7–5.6) | 6.7%(2.6–15.9) |
| 80 | *APH(4)-Ia* |  | 2.6%(1.0–6.5) | 5.0%(1.7–13.7) |
| 81 | *aac(3)-IV* |  | 2.6%(1.0–6.5) | 5.0%(1.7–13.7) |
| 82 | *LAP-2* |  | 3.9%(1.8–8.3) | 1.7%(0.3–8.9) |
| 83 | *TEM-135* |  | 2.0%(0.7–5.6) | 6.7%(2.6–15.9) |
| 84 | *TEM-57* |  | 2.0%(0.7–5.6) | 6.7%(2.6–15.9) |
| 85 | *Salmonella enterica gyrA with mutation conferring resistance to triclosan* |  | 3.9%(1.8–8.3) | 0.0%(0.0–6.0) |
| 86 | *catII from E.coli K-12* |  | 1.3%(0.4–4.6) | 6.7%(2.6–15.9) |
| 87 | *aadA7* |  | 3.3%(1.4–7.4) | 0.0%(0.0–6.0) |
| 88 | *acrD* |  | 2.6%(1.0–6.5) | 1.7%(0.3–8.9) |
| 89 | *qacE* |  | 3.3%(1.4–7.4) | 0.0%(0.0–6.0) |
| 90 | *aac(3)-Id* |  | 3.3%(1.4–7.4) | 0.0%(0.0–6.0) |
| 91 | *E.coli parC conferring resistance to fluoroquinolones* |  | 3.3%(1.4–7.4) | 0.0%(0.0–6.0) |
| 92 | *QnrB10* |  | 2.0%(0.7–5.6) | 1.7%(0.3–8.9) |
| 93 | *aac(3)-IIe* |  | 2.0%(0.7–5.6) | 1.7%(0.3–8.9) |
| 94 | *FosA3* |  | 2.6%(1.0–6.5) | 0.0%(0.0–6.0) |
| 95 | *DHA-1* |  | 2.6%(1.0–6.5) | 0.0%(0.0–6.0) |
| 96 | *QnrB4* |  | 2.6%(1.0–6.5) | 0.0%(0.0–6.0) |
| 97 | *aadA3* |  | 1.3%(0.4–4.6) | 3.3%(0.9–11.4) |
| 98 | *dfrA1* |  | 2.6%(1.0–6.5) | 0.0%(0.0–6.0) |
| 99 | *QnrS2* |  | 0.7%(0.1–3.6) | 3.3%(0.9–11.4) |
| 100 | *CMY-2* |  | 2.0%(0.7–5.6) | 0.0%(0.0–6.0) |
| 101 | *TEM-90* |  | 2.0%(0.7–5.6) | 0.0%(0.0–6.0) |
| 102 | *rmtB* |  | 2.0%(0.7–5.6) | 0.0%(0.0–6.0) |
| 103 | *TEM-150* |  | 2.0%(0.7–5.6) | 0.0%(0.0–6.0) |
| 104 | *AcrF* |  | 1.3%(0.4–4.6) | 0.0%(0.0–6.0) |
| 105 | *APH(3')-IIa* |  | 1.3%(0.4–4.6) | 0.0%(0.0–6.0) |
| 106 | *aadA9* |  | 0.0%(0.0–2.4) | 3.3%(0.9–11.4) |
| 107 | *QepA2* |  | 1.3%(0.4–4.6) | 0.0%(0.0–6.0) |
| 108 | *ErmB* |  | 1.3%(0.4–4.6) | 0.0%(0.0–6.0) |
| 109 | *TEM-216* |  | 1.3%(0.4–4.6) | 0.0%(0.0–6.0) |
| 110 | *tet(D)* |  | 1.3%(0.4–4.6) | 0.0%(0.0–6.0) |
| 111 | *CARB-3* |  | 1.3%(0.4–4.6) | 0.0%(0.0–6.0) |
| 112 | *aadA22* |  | 1.3%(0.4–4.6) | 0.0%(0.0–6.0) |
| 113 | *aadA24* |  | 1.3%(0.4–4.6) | 0.0%(0.0–6.0) |
| 114 | *aadA12* |  | 1.3%(0.4–4.6) | 0.0%(0.0–6.0) |
| 115 | *QnrD1* |  | 0.7%(0.1–3.6) | 0.0%(0.0–6.0) |
| 116 | *CTX-M-64* |  | 0.7%(0.1–3.6) | 0.0%(0.0–6.0) |
| 117 | *msrE* |  | 0.7%(0.1–3.6) | 0.0%(0.0–6.0) |
| 118 | *mphE* |  | 0.7%(0.1–3.6) | 0.0%(0.0–6.0) |
| 119 | *aadA25* |  | 0.7%(0.1–3.6) | 0.0%(0.0–6.0) |
| 120 | *CTX-M-27* |  | 0.7%(0.1–3.6) | 0.0%(0.0–6.0) |
| 121 | *aadA23* |  | 0.7%(0.1–3.6) | 0.0%(0.0–6.0) |
| 122 | *aac(3)-IIc* |  | 0.7%(0.1–3.6) | 0.0%(0.0–6.0) |
| 123 | *CTX-M-65* |  | 0.7%(0.1–3.6) | 0.0%(0.0–6.0) |
| 124 | *CTX-M-161* |  | 0.7%(0.1–3.6) | 0.0%(0.0–6.0) |
| 125 | *CTX-M-123* |  | 0.7%(0.1–3.6) | 0.0%(0.0–6.0) |
| 126 | *CTX-M-134* |  | 0.7%(0.1–3.6) | 0.0%(0.0–6.0) |
| 127 | *TEM-208* |  | 0.0%(0.0–2.4) | 1.7%(0.3–8.9) |
| 128 | *QnrA1* |  | 0.7%(0.1–3.6) | 0.0%(0.0–6.0) |

*, *P*<0.05

**Table S2 Comparative analysis of virulence genes between Human and Pork (n=213)**

| No | VFDB |  | Human(%(95%CI)) (n=153) | Pork(%(95%CI)) (n=60) |
| --- | --- | --- | --- | --- |
| 1 | *sciJ* | * | 68.0%(60.2–74.8) | 98.3%(91.1–99.7) |
| 2 | *csgD* | * | 70.6%(62.9–77.2) | 26.7%(17.1–39.0) |
| 3 | *hemE* | * | 71.2%(63.6–77.8) | 41.7%(30.1–54.3) |
| 4 | *sciS/icmF* | * | 74.5%(67.1–80.8) | 98.3%(91.1–99.7) |
| 5 | *sciQ* | * | 74.5%(67.1–80.8) | 98.3%(91.1–99.7) |
| 6 | *sciP* | * | 74.5%(67.1–80.8) | 98.3%(91.1–99.7) |
| 7 | *STM0278* | * | 74.5%(67.1–80.8) | 98.3%(91.1–99.7) |
| 8 | *sciL* | * | 74.5%(67.1–80.8) | 98.3%(91.1–99.7) |
| 9 | *sciT* | * | 75.2%(67.8–81.3) | 98.3%(91.1–99.7) |
| 10 | *sciO* | * | 75.8%(68.5–81.9) | 98.3%(91.1–99.7) |
| 11 | *sciN* | * | 75.8%(68.5–81.9) | 98.3%(91.1–99.7) |
| 12 | *sciI* | * | 75.8%(68.5–81.9) | 98.3%(91.1–99.7) |
| 13 | *sciH* | * | 75.8%(68.5–81.9) | 98.3%(91.1–99.7) |
| 14 | *sciE* | * | 75.8%(68.5–81.9) | 98.3%(91.1–99.7) |
| 15 | *sciD* | * | 75.8%(68.5–81.9) | 98.3%(91.1–99.7) |
| 16 | *sciC* | * | 75.8%(68.5–81.9) | 98.3%(91.1–99.7) |
| 17 | *clpV* | * | 76.5%(69.2–82.5) | 98.3%(91.1–99.7) |
| 18 | *cdtB* | * | 20.3%(14.7–27.3) | 1.7%(0.3–8.9) |
| 19 | *pltA* | * | 20.3%(14.7–27.3) | 1.7%(0.3–8.9) |
| 20 | *inv* | * | 51.0%(43.1–58.8) | 75.0%(62.8–84.2) |
| 21 | *sciF* | * | 81.7%(74.8–87.0) | 98.3%(91.1–99.7) |
| 22 | *tcfD* | * | 17.6%(12.4–24.5) | 1.7%(0.3–8.9) |
| 23 | *sopD2* | * | 85.6%(79.2–90.3) | 100.0%(94.0–100.0) |
| 24 | *allC* | * | 85.6%(79.2–90.3) | 100.0%(94.0–100.0) |
| 25 | *sciV* | * | 85.6%(79.2–90.3) | 100.0%(94.0–100.0) |
| 26 | *sciU* | * | 86.3%(79.9–90.8) | 100.0%(94.0–100.0) |
| 27 | *safC* | * | 94.8%(90.0–97.3) | 81.7%(70.1–89.4) |
| 28 | *safB* | * | 94.8%(90.0–97.3) | 81.7%(70.1–89.4) |
| 29 | *sciA* | * | 90.2%(84.5–94.0) | 100.0%(94.0–100.0) |
| 30 | *sciB* | * | 90.2%(84.5–94.0) | 100.0%(94.0–100.0) |
| 31 | *vgrS* | * | 90.2%(84.5–94.0) | 100.0%(94.0–100.0) |
| 32 | *allD* | * | 86.9%(80.7–91.4) | 100.0%(94.0–100.0) |
| 33 | *allR* | * | 86.9%(80.7–91.4) | 100.0%(94.0–100.0) |
| 34 | *allB* | * | 86.9%(80.7–91.4) | 100.0%(94.0–100.0) |
| 35 | *sefR* | * | 15.7%(10.8–22.3) | 1.7%(0.3–8.9) |
| 36 | *sefD* | * | 15.7%(10.8–22.3) | 1.7%(0.3–8.9) |
| 37 | *sefC* | * | 15.7%(10.8–22.3) | 1.7%(0.3–8.9) |
| 38 | *sefB* | * | 15.7%(10.8–22.3) | 1.7%(0.3–8.9) |
| 39 | *safD* | * | 94.1%(89.2–96.9) | 81.7%(70.1–89.4) |
| 40 | *SG1030* | * | 15.0%(10.2–21.5) | 1.7%(0.3–8.9) |
| 41 | *SG1031* | * | 15.0%(10.2–21.5) | 1.7%(0.3–8.9) |
| 42 | *mig-5* | * | 17.6%(12.4–24.5) | 3.3%(0.9–11.4) |
| 43 | *spvR* | * | 17.6%(12.4–24.5) | 3.3%(0.9–11.4) |
| 44 | *spvA* | * | 17.6%(12.4–24.5) | 3.3%(0.9–11.4) |
| 45 | *spvB* | * | 17.6%(12.4–24.5) | 3.3%(0.9–11.4) |
| 46 | *spvC* | * | 17.6%(12.4–24.5) | 3.3%(0.9–11.4) |
| 47 | *spvD* | * | 17.6%(12.4–24.5) | 3.3%(0.9–11.4) |
| 48 | *stfC* | * | 88.2%(82.2–92.4) | 100.0%(94.0–100.0) |
| 49 | *stfG* | * | 88.2%(82.2–92.4) | 100.0%(94.0–100.0) |
| 50 | *stfF* | * | 88.2%(82.2–92.4) | 100.0%(94.0–100.0) |
| 51 | *stfE* | * | 88.2%(82.2–92.4) | 100.0%(94.0–100.0) |
| 52 | *stfD* | * | 88.2%(82.2–92.4) | 100.0%(94.0–100.0) |
| 53 | *stfA* | * | 88.2%(82.2–92.4) | 100.0%(94.0–100.0) |
| 54 | *pltB* | * | 11.8%(7.6–17.8) | 0.0%(0.0–6.0) |
| 55 | *sefA* | * | 14.4%(9.7–20.8) | 1.7%(0.3–8.9) |
| 56 | *SG1029* | * | 14.4%(9.7–20.8) | 1.7%(0.3–8.9) |
| 57 | *pefB* | * | 17.0%(11.9–23.7) | 3.3%(0.9–11.4) |
| 58 | *pefA* | * | 17.0%(11.9–23.7) | 3.3%(0.9–11.4) |
| 59 | *cheB* | * | 56.2%(48.3–63.8) | 36.7%(25.6–49.3) |
| 60 | *fepB* | * | 11.1%(7.1–17.1) | 25.0%(15.8–37.2) |
| 61 | *sseK1* | * | 83.7%(77.0–88.7) | 96.7%(88.6–99.1) |
| 62 | *pefD* | * | 16.3%(11.3–23.0) | 3.3%(0.9–11.4) |
| 63 | *pefC* | * | 16.3%(11.3–23.0) | 3.3%(0.9–11.4) |
| 64 | *ABZJ_00085* | * | 13.7%(9.2–20.1) | 28.3%(18.5–40.8) |
| 65 | *fljA* | * | 64.7%(56.9–71.8) | 46.7%(34.6–59.1) |
| 66 | *rck* | * | 13.1%(8.6–19.3) | 1.7%(0.3–8.9) |
| 67 | *SPA1306* | * | 13.1%(8.6–19.3) | 1.7%(0.3–8.9) |
| 68 | *sipA* | * | 75.2%(67.8–81.3) | 58.3%(45.7–69.9) |
| 69 | *sipA/sspA* | * | 24.8%(18.7–32.2) | 41.7%(30.1–54.3) |
| 70 | *cshB* | * | 15.7%(10.8–22.3) | 3.3%(0.9–11.4) |
| 71 | *faeC* | * | 15.7%(10.8–22.3) | 3.3%(0.9–11.4) |
| 72 | *tar/cheM* | * | 88.2%(82.2–92.4) | 75.0%(62.8–84.2) |
| 73 | *ehaB* | * | 63.4%(55.5–70.6) | 80.0%(68.2–88.2) |
| 74 | *clpE* | * | 12.4%(8.1–18.6) | 1.7%(0.3–8.9) |
| 75 | *STY1498* | * | 7.2%(4.1–12.4) | 0.0%(0.0–6.0) |
| 76 | *ddhA* | * | 63.4%(55.5–70.6) | 46.7%(34.6–59.1) |
| 77 | *ddhC* | * | 63.4%(55.5–70.6) | 46.7%(34.6–59.1) |
| 78 | *sciK* |  | 64.1%(56.2–71.2) | 78.3%(66.4–86.9) |
| 79 | *sciR* |  | 66.7%(58.9–73.6) | 80.0%(68.2–88.2) |
| 80 | *steB* |  | 63.4%(55.5–70.6) | 50.0%(37.7–62.3) |
| 81 | *ssaN* |  | 88.2%(82.2–92.4) | 96.7%(88.6–99.1) |
| 82 | *gtrB* |  | 86.9%(80.7–91.4) | 76.7%(64.6–85.6) |
| 83 | *steD* |  | 62.7%(54.9–70.0) | 50.0%(37.7–62.3) |
| 84 | *steE* |  | 62.7%(54.9–70.0) | 50.0%(37.7–62.3) |
| 85 | *steF* |  | 62.7%(54.9–70.0) | 50.0%(37.7–62.3) |
| 86 | *gtrA* |  | 75.8%(68.5–81.9) | 65.0%(52.4–75.8) |
| 87 | *tcfC* |  | 32.0%(25.2–39.8) | 21.7%(13.1–33.6) |
| 88 | *tcfB* |  | 32.0%(25.2–39.8) | 21.7%(13.1–33.6) |
| 89 | *shdA* |  | 80.4%(73.4–85.9) | 71.7%(59.2–81.5) |
| 90 | *safA* |  | 60.1%(52.2–67.5) | 50.0%(37.7–62.3) |
| 91 | *tcfA* |  | 30.7%(24.0–38.4) | 21.7%(13.1–33.6) |
| 92 | *stdD* |  | 45.1%(37.4–53.0) | 55.0%(42.5–66.9) |
| 93 | *staA* |  | 28.8%(22.2–36.4) | 20.0%(11.8–31.8) |
| 94 | *staB* |  | 28.8%(22.2–36.4) | 20.0%(11.8–31.8) |
| 95 | *staC* |  | 28.8%(22.2–36.4) | 20.0%(11.8–31.8) |
| 96 | *staD* |  | 28.8%(22.2–36.4) | 20.0%(11.8–31.8) |
| 97 | *staE* |  | 28.8%(22.2–36.4) | 20.0%(11.8–31.8) |
| 98 | *staF* |  | 28.8%(22.2–36.4) | 20.0%(11.8–31.8) |
| 99 | *staG* |  | 28.8%(22.2–36.4) | 20.0%(11.8–31.8) |
| 100 | *iroB* |  | 100.0%(97.6–100.0) | 98.3%(91.1–99.7) |
| 101 | *iroC* |  | 100.0%(97.6–100.0) | 98.3%(91.1–99.7) |
| 102 | *iroD* |  | 100.0%(97.6–100.0) | 98.3%(91.1–99.7) |
| 103 | *iroE* |  | 100.0%(97.6–100.0) | 98.3%(91.1–99.7) |
| 104 | *iroN* |  | 100.0%(97.6–100.0) | 98.3%(91.1–99.7) |
| 105 | *mig-14* |  | 100.0%(97.6–100.0) | 98.3%(91.1–99.7) |
| 106 | *fepG* |  | 86.9%(80.7–91.4) | 80.0%(68.2–88.2) |
| 107 | *fliD* |  | 71.9%(64.3–78.4) | 63.3%(50.7–74.4) |
| 108 | *sspH2* |  | 24.2%(18.1–31.5) | 31.7%(21.3–44.2) |
| 109 | *stcC* |  | 38.6%(31.2–46.5) | 46.7%(34.6–59.1) |
| 110 | *stcA* |  | 38.6%(31.2–46.5) | 46.7%(34.6–59.1) |
| 111 | *stcB* |  | 38.6%(31.2–46.5) | 46.7%(34.6–59.1) |
| 112 | *stcD* |  | 38.6%(31.2–46.5) | 46.7%(34.6–59.1) |
| 113 | *ompD* |  | 58.2%(50.2–65.7) | 50.0%(37.7–62.3) |
| 114 | *stkG* |  | 21.6%(15.8–28.7) | 28.3%(18.5–40.8) |
| 115 | *stkF* |  | 21.6%(15.8–28.7) | 28.3%(18.5–40.8) |
| 116 | *stkE* |  | 21.6%(15.8–28.7) | 28.3%(18.5–40.8) |
| 117 | *stkD* |  | 21.6%(15.8–28.7) | 28.3%(18.5–40.8) |
| 118 | *stkC* |  | 21.6%(15.8–28.7) | 28.3%(18.5–40.8) |
| 119 | *stkB* |  | 21.6%(15.8–28.7) | 28.3%(18.5–40.8) |
| 120 | *stkA* |  | 21.6%(15.8–28.7) | 28.3%(18.5–40.8) |
| 121 | *SeAg_B4896* |  | 11.1%(7.1–17.1) | 16.7%(9.3–28.0) |
| 122 | *avrA* |  | 86.3%(79.9–90.8) | 91.7%(81.9–96.4) |
| 123 | *stjB* |  | 43.1%(35.6–51.1) | 50.0%(37.7–62.3) |
| 124 | *stjA* |  | 43.1%(35.6–51.1) | 50.0%(37.7–62.3) |
| 125 | *lpfE* |  | 67.3%(59.5–74.2) | 73.3%(61.0–82.9) |
| 126 | *lpfD* |  | 67.3%(59.5–74.2) | 73.3%(61.0–82.9) |
| 127 | *lpfC* |  | 67.3%(59.5–74.2) | 73.3%(61.0–82.9) |
| 128 | *lpfB* |  | 67.3%(59.5–74.2) | 73.3%(61.0–82.9) |
| 129 | *lpfA* |  | 67.3%(59.5–74.2) | 73.3%(61.0–82.9) |
| 130 | *gogB* |  | 13.7%(9.2–20.1) | 18.3%(10.6–29.9) |
| 131 | *fljB* |  | 40.5%(33.1–48.4) | 35.0%(24.2–47.6) |
| 132 | *sipC/sspC* |  | 98.0%(94.4–99.3) | 100.0%(94.0–100.0) |
| 133 | *galF* |  | 98.0%(94.4–99.3) | 100.0%(94.0–100.0) |
| 134 | *stiA* |  | 98.0%(94.4–99.3) | 100.0%(94.0–100.0) |
| 135 | *stiB* |  | 98.0%(94.4–99.3) | 100.0%(94.0–100.0) |
| 136 | *stiC* |  | 98.0%(94.4–99.3) | 100.0%(94.0–100.0) |
| 137 | *stiH* |  | 98.0%(94.4–99.3) | 100.0%(94.0–100.0) |
| 138 | *N559_1747* |  | 2.0%(0.7–5.6) | 0.0%(0.0–6.0) |
| 139 | *sinH* |  | 97.4%(93.5–99.0) | 100.0%(94.0–100.0) |
| 140 | *stbC* |  | 97.4%(93.5–99.0) | 100.0%(94.0–100.0) |
| 141 | *sciM* |  | 26.8%(20.4–34.3) | 31.7%(21.3–44.2) |
| 142 | *tcpC* |  | 3.9%(1.8–8.3) | 1.7%(0.3–8.9) |
| 143 | *sseI/srfH* |  | 39.2%(31.8–47.1) | 43.3%(31.6–55.9) |
| 144 | *sciW* |  | 47.7%(40.0–55.6) | 51.7%(39.3–63.8) |
| 145 | *stjC* |  | 43.1%(35.6–51.1) | 46.7%(34.6–59.1) |
| 146 | *fliC* |  | 34.6%(27.6–42.5) | 31.7%(21.3–44.2) |
| 147 | *sopE* |  | 32.7%(25.8–40.5) | 30.0%(19.9–42.5) |
| 148 | *sspH1* |  | 13.7%(9.2–20.1) | 11.7%(5.8–22.2) |
| 149 | *sipB/sspB* |  | 51.0%(43.1–58.8) | 53.3%(40.9–65.4) |
| 150 | *STM4574* |  | 32.0%(25.2–39.8) | 30.0%(19.9–42.5) |
| 151 | *STM4575* |  | 28.1%(21.6–35.7) | 30.0%(19.9–42.5) |
| 152 | *SeAg_B4897* |  | 15.0%(10.2–21.5) | 16.7%(9.3–28.0) |
| 153 | *sipB* |  | 48.4%(40.6–56.2) | 46.7%(34.6–59.1) |
| 154 | *sseK2* |  | 68.0%(60.2–74.8) | 66.7%(54.1–77.3) |
| 155 | *pegD* |  | 43.8%(36.2–51.7) | 45.0%(33.1–57.5) |
| 156 | *pegC* |  | 43.8%(36.2–51.7) | 45.0%(33.1–57.5) |
| 157 | *pegB* |  | 43.8%(36.2–51.7) | 45.0%(33.1–57.5) |
| 158 | *pegA* |  | 43.8%(36.2–51.7) | 45.0%(33.1–57.5) |
| 159 | *ratB* |  | 100.0%(97.6–100.0) | 100.0%(94.0–100.0) |
| 160 | *steA* |  | 100.0%(97.6–100.0) | 100.0%(94.0–100.0) |
| 161 | *steC* |  | 100.0%(97.6–100.0) | 100.0%(94.0–100.0) |
| 162 | *sopA* |  | 100.0%(97.6–100.0) | 100.0%(94.0–100.0) |
| 163 | *stbD* |  | 99.3%(96.4–99.9) | 100.0%(94.0–100.0) |
| 164 | *hilC* |  | 100.0%(97.6–100.0) | 100.0%(94.0–100.0) |
| 165 | *acrB* |  | 100.0%(97.6–100.0) | 100.0%(94.0–100.0) |
| 166 | *sseF* |  | 100.0%(97.6–100.0) | 100.0%(94.0–100.0) |
| 167 | *sptP* |  | 100.0%(97.6–100.0) | 100.0%(94.0–100.0) |
| 168 | *ssaV* |  | 100.0%(97.6–100.0) | 100.0%(94.0–100.0) |
| 169 | *sseC* |  | 100.0%(97.6–100.0) | 100.0%(94.0–100.0) |
| 170 | *ipaH* |  | 100.0%(97.6–100.0) | 100.0%(94.0–100.0) |
| 171 | *invF* |  | 100.0%(97.6–100.0) | 100.0%(94.0–100.0) |
| 172 | *fimD* |  | 100.0%(97.6–100.0) | 100.0%(94.0–100.0) |
| 173 | *gmd* |  | 99.3%(96.4–99.9) | 100.0%(94.0–100.0) |
| 174 | *misL* |  | 99.3%(96.4–99.9) | 100.0%(94.0–100.0) |
| 175 | *sthE* |  | 100.0%(97.6–100.0) | 100.0%(94.0–100.0) |
| 176 | *sseL* |  | 100.0%(97.6–100.0) | 100.0%(94.0–100.0) |
| 177 | *sopE2* |  | 100.0%(97.6–100.0) | 100.0%(94.0–100.0) |
| 178 | *kdsA* |  | 100.0%(97.6–100.0) | 100.0%(94.0–100.0) |
| 179 | *entB* |  | 100.0%(97.6–100.0) | 100.0%(94.0–100.0) |
| 180 | *ssaU* |  | 100.0%(97.6–100.0) | 100.0%(94.0–100.0) |
| 181 | *ssaT* |  | 100.0%(97.6–100.0) | 100.0%(94.0–100.0) |
| 182 | *ssaS* |  | 100.0%(97.6–100.0) | 100.0%(94.0–100.0) |
| 183 | *ssaR* |  | 100.0%(97.6–100.0) | 100.0%(94.0–100.0) |
| 184 | *ssaQ* |  | 100.0%(97.6–100.0) | 100.0%(94.0–100.0) |
| 185 | *ssaP* |  | 100.0%(97.6–100.0) | 100.0%(94.0–100.0) |
| 186 | *ssaO* |  | 100.0%(97.6–100.0) | 100.0%(94.0–100.0) |
| 187 | *ssaM* |  | 100.0%(97.6–100.0) | 100.0%(94.0–100.0) |
| 188 | *ssaL* |  | 100.0%(97.6–100.0) | 100.0%(94.0–100.0) |
| 189 | *ssaK* |  | 100.0%(97.6–100.0) | 100.0%(94.0–100.0) |
| 190 | *ssaJ* |  | 100.0%(97.6–100.0) | 100.0%(94.0–100.0) |
| 191 | *ssaI* |  | 100.0%(97.6–100.0) | 100.0%(94.0–100.0) |
| 192 | *ssaH* |  | 100.0%(97.6–100.0) | 100.0%(94.0–100.0) |
| 193 | *ssaG* |  | 100.0%(97.6–100.0) | 100.0%(94.0–100.0) |
| 194 | *sseG* |  | 100.0%(97.6–100.0) | 100.0%(94.0–100.0) |
| 195 | *sscB* |  | 100.0%(97.6–100.0) | 100.0%(94.0–100.0) |
| 196 | *sseE* |  | 100.0%(97.6–100.0) | 100.0%(94.0–100.0) |
| 197 | *sseD* |  | 100.0%(97.6–100.0) | 100.0%(94.0–100.0) |
| 198 | *sscA* |  | 100.0%(97.6–100.0) | 100.0%(94.0–100.0) |
| 199 | *sseB* |  | 100.0%(97.6–100.0) | 100.0%(94.0–100.0) |
| 200 | *sseA* |  | 100.0%(97.6–100.0) | 100.0%(94.0–100.0) |
| 201 | *ssaD* |  | 100.0%(97.6–100.0) | 100.0%(94.0–100.0) |
| 202 | *ssaC* |  | 100.0%(97.6–100.0) | 100.0%(94.0–100.0) |
| 203 | *spiC/ssaB* |  | 100.0%(97.6–100.0) | 100.0%(94.0–100.0) |
| 204 | *ssrA* |  | 100.0%(97.6–100.0) | 100.0%(94.0–100.0) |
| 205 | *ssrB* |  | 100.0%(97.6–100.0) | 100.0%(94.0–100.0) |
| 206 | *sitC* |  | 100.0%(97.6–100.0) | 100.0%(94.0–100.0) |
| 207 | *sprB* |  | 100.0%(97.6–100.0) | 100.0%(94.0–100.0) |
| 208 | *orgC* |  | 100.0%(97.6–100.0) | 100.0%(94.0–100.0) |
| 209 | *orgB* |  | 100.0%(97.6–100.0) | 100.0%(94.0–100.0) |
| 210 | *orgA* |  | 100.0%(97.6–100.0) | 100.0%(94.0–100.0) |
| 211 | *prgK* |  | 100.0%(97.6–100.0) | 100.0%(94.0–100.0) |
| 212 | *prgJ* |  | 100.0%(97.6–100.0) | 100.0%(94.0–100.0) |
| 213 | *prgI* |  | 100.0%(97.6–100.0) | 100.0%(94.0–100.0) |
| 214 | *prgH* |  | 100.0%(97.6–100.0) | 100.0%(94.0–100.0) |
| 215 | *hilD* |  | 100.0%(97.6–100.0) | 100.0%(94.0–100.0) |
| 216 | *hilA* |  | 100.0%(97.6–100.0) | 100.0%(94.0–100.0) |
| 217 | *iagB* |  | 100.0%(97.6–100.0) | 100.0%(94.0–100.0) |
| 218 | *sicP* |  | 100.0%(97.6–100.0) | 100.0%(94.0–100.0) |
| 219 | *invH* |  | 100.0%(97.6–100.0) | 100.0%(94.0–100.0) |
| 220 | *rpoS* |  | 100.0%(97.6–100.0) | 100.0%(94.0–100.0) |
| 221 | *sopD* |  | 100.0%(97.6–100.0) | 100.0%(94.0–100.0) |
| 222 | *KOX_12990* |  | 100.0%(97.6–100.0) | 100.0%(94.0–100.0) |
| 223 | *fimA* |  | 100.0%(97.6–100.0) | 100.0%(94.0–100.0) |
| 224 | *fimI* |  | 100.0%(97.6–100.0) | 100.0%(94.0–100.0) |
| 225 | *fimC* |  | 100.0%(97.6–100.0) | 100.0%(94.0–100.0) |
| 226 | *fimH* |  | 100.0%(97.6–100.0) | 100.0%(94.0–100.0) |
| 227 | *fimF* |  | 100.0%(97.6–100.0) | 100.0%(94.0–100.0) |
| 228 | *fimZ* |  | 100.0%(97.6–100.0) | 100.0%(94.0–100.0) |
| 229 | *fimY* |  | 100.0%(97.6–100.0) | 100.0%(94.0–100.0) |
| 230 | *fimW* |  | 100.0%(97.6–100.0) | 100.0%(94.0–100.0) |
| 231 | *STM0570* |  | 100.0%(97.6–100.0) | 100.0%(94.0–100.0) |
| 232 | *fepC* |  | 100.0%(97.6–100.0) | 100.0%(94.0–100.0) |
| 233 | *fepD* |  | 100.0%(97.6–100.0) | 100.0%(94.0–100.0) |
| 234 | *entC* |  | 100.0%(97.6–100.0) | 100.0%(94.0–100.0) |
| 235 | *entE* |  | 100.0%(97.6–100.0) | 100.0%(94.0–100.0) |
| 236 | *entA* |  | 100.0%(97.6–100.0) | 100.0%(94.0–100.0) |
| 237 | *fur* |  | 100.0%(97.6–100.0) | 100.0%(94.0–100.0) |
| 238 | *gnd* |  | 100.0%(97.6–100.0) | 100.0%(94.0–100.0) |
| 239 | *fliR* |  | 100.0%(97.6–100.0) | 100.0%(94.0–100.0) |
| 240 | *fliQ* |  | 100.0%(97.6–100.0) | 100.0%(94.0–100.0) |
| 241 | *fliP* |  | 100.0%(97.6–100.0) | 100.0%(94.0–100.0) |
| 242 | *fliO* |  | 100.0%(97.6–100.0) | 100.0%(94.0–100.0) |
| 243 | *fliN* |  | 100.0%(97.6–100.0) | 100.0%(94.0–100.0) |
| 244 | *fliM* |  | 100.0%(97.6–100.0) | 100.0%(94.0–100.0) |
| 245 | *fliL* |  | 100.0%(97.6–100.0) | 100.0%(94.0–100.0) |
| 246 | *fliK* |  | 100.0%(97.6–100.0) | 100.0%(94.0–100.0) |
| 247 | *fliJ* |  | 100.0%(97.6–100.0) | 100.0%(94.0–100.0) |
| 248 | *fliI* |  | 100.0%(97.6–100.0) | 100.0%(94.0–100.0) |
| 249 | *fliH* |  | 100.0%(97.6–100.0) | 100.0%(94.0–100.0) |
| 250 | *fliG* |  | 100.0%(97.6–100.0) | 100.0%(94.0–100.0) |
| 251 | *fliF* |  | 100.0%(97.6–100.0) | 100.0%(94.0–100.0) |
| 252 | *fliE* |  | 100.0%(97.6–100.0) | 100.0%(94.0–100.0) |
| 253 | *fliT* |  | 100.0%(97.6–100.0) | 100.0%(94.0–100.0) |
| 254 | *fliS* |  | 100.0%(97.6–100.0) | 100.0%(94.0–100.0) |
| 255 | *fliB* |  | 100.0%(97.6–100.0) | 100.0%(94.0–100.0) |
| 256 | *fliA* |  | 100.0%(97.6–100.0) | 100.0%(94.0–100.0) |
| 257 | *fliZ* |  | 100.0%(97.6–100.0) | 100.0%(94.0–100.0) |
| 258 | *fliY* |  | 100.0%(97.6–100.0) | 100.0%(94.0–100.0) |
| 259 | *flhD* |  | 100.0%(97.6–100.0) | 100.0%(94.0–100.0) |
| 260 | *flhC* |  | 100.0%(97.6–100.0) | 100.0%(94.0–100.0) |
| 261 | *motA* |  | 100.0%(97.6–100.0) | 100.0%(94.0–100.0) |
| 262 | *motB* |  | 100.0%(97.6–100.0) | 100.0%(94.0–100.0) |
| 263 | *cheA* |  | 100.0%(97.6–100.0) | 100.0%(94.0–100.0) |
| 264 | *cheW* |  | 100.0%(97.6–100.0) | 100.0%(94.0–100.0) |
| 265 | *cheR* |  | 100.0%(97.6–100.0) | 100.0%(94.0–100.0) |
| 266 | *cheY* |  | 100.0%(97.6–100.0) | 100.0%(94.0–100.0) |
| 267 | *cheZ* |  | 100.0%(97.6–100.0) | 100.0%(94.0–100.0) |
| 268 | *flhB* |  | 100.0%(97.6–100.0) | 100.0%(94.0–100.0) |
| 269 | *flhA* |  | 100.0%(97.6–100.0) | 100.0%(94.0–100.0) |
| 270 | *flhE* |  | 100.0%(97.6–100.0) | 100.0%(94.0–100.0) |
| 271 | *slrP* |  | 99.3%(96.4–99.9) | 100.0%(94.0–100.0) |
| 272 | *ppdD* |  | 100.0%(97.6–100.0) | 100.0%(94.0–100.0) |
| 273 | *Fphi_1039* |  | 100.0%(97.6–100.0) | 100.0%(94.0–100.0) |
| 274 | *ECS88_3547* |  | 100.0%(97.6–100.0) | 100.0%(94.0–100.0) |
| 275 | *mgtB* |  | 100.0%(97.6–100.0) | 100.0%(94.0–100.0) |
| 276 | *phoP* |  | 100.0%(97.6–100.0) | 100.0%(94.0–100.0) |
| 277 | *phoQ* |  | 100.0%(97.6–100.0) | 100.0%(94.0–100.0) |
| 278 | *sifA* |  | 100.0%(97.6–100.0) | 100.0%(94.0–100.0) |
| 279 | *flgL* |  | 100.0%(97.6–100.0) | 100.0%(94.0–100.0) |
| 280 | *flgK* |  | 100.0%(97.6–100.0) | 100.0%(94.0–100.0) |
| 281 | *flgJ* |  | 100.0%(97.6–100.0) | 100.0%(94.0–100.0) |
| 282 | *flgI* |  | 100.0%(97.6–100.0) | 100.0%(94.0–100.0) |
| 283 | *flgH* |  | 100.0%(97.6–100.0) | 100.0%(94.0–100.0) |
| 284 | *flgG* |  | 100.0%(97.6–100.0) | 100.0%(94.0–100.0) |
| 285 | *flgF* |  | 100.0%(97.6–100.0) | 100.0%(94.0–100.0) |
| 286 | *flgE* |  | 100.0%(97.6–100.0) | 100.0%(94.0–100.0) |
| 287 | *flgD* |  | 100.0%(97.6–100.0) | 100.0%(94.0–100.0) |
| 288 | *flgC* |  | 100.0%(97.6–100.0) | 100.0%(94.0–100.0) |
| 289 | *flgB* |  | 100.0%(97.6–100.0) | 100.0%(94.0–100.0) |
| 290 | *flgA* |  | 100.0%(97.6–100.0) | 100.0%(94.0–100.0) |
| 291 | *flgM* |  | 100.0%(97.6–100.0) | 100.0%(94.0–100.0) |
| 292 | *flgN* |  | 100.0%(97.6–100.0) | 100.0%(94.0–100.0) |
| 293 | *csgC* |  | 100.0%(97.6–100.0) | 100.0%(94.0–100.0) |
| 294 | *csgE* |  | 100.0%(97.6–100.0) | 100.0%(94.0–100.0) |
| 295 | *csgF* |  | 100.0%(97.6–100.0) | 100.0%(94.0–100.0) |
| 296 | *csgG* |  | 100.0%(97.6–100.0) | 100.0%(94.0–100.0) |
| 297 | *sopB/sigD* |  | 100.0%(97.6–100.0) | 100.0%(94.0–100.0) |
| 298 | *pipB* |  | 100.0%(97.6–100.0) | 100.0%(94.0–100.0) |
| 299 | *Z1307* |  | 100.0%(97.6–100.0) | 100.0%(94.0–100.0) |
| 300 | *ibeC* |  | 100.0%(97.6–100.0) | 100.0%(94.0–100.0) |
| 301 | *bcfC* |  | 98.7%(95.4–99.6) | 98.3%(91.1–99.7) |
| 302 | *sthA* |  | 99.3%(96.4–99.9) | 100.0%(94.0–100.0) |
| 303 | *sthB* |  | 100.0%(97.6–100.0) | 100.0%(94.0–100.0) |
| 304 | *sthC* |  | 100.0%(97.6–100.0) | 100.0%(94.0–100.0) |
| 305 | *sthD* |  | 100.0%(97.6–100.0) | 100.0%(94.0–100.0) |
| 306 | *A225_4123* |  | 100.0%(97.6–100.0) | 100.0%(94.0–100.0) |
| 307 | *siiE* |  | 100.0%(97.6–100.0) | 100.0%(94.0–100.0) |
| 308 | *pagN* |  | 100.0%(97.6–100.0) | 100.0%(94.0–100.0) |
| 309 | *flk* |  | 100.0%(97.6–100.0) | 100.0%(94.0–100.0) |
| 310 | *KOX_00005* |  | 100.0%(97.6–100.0) | 100.0%(94.0–100.0) |
| 311 | *mgtC* |  | 100.0%(97.6–100.0) | 100.0%(94.0–100.0) |
| 312 | *sifB* |  | 99.3%(96.4–99.9) | 100.0%(94.0–100.0) |
| 313 | *ssaE* |  | 99.3%(96.4–99.9) | 100.0%(94.0–100.0) |
| 314 | *iacP* |  | 99.3%(96.4–99.9) | 100.0%(94.0–100.0) |
| 315 | *sipD* |  | 99.3%(96.4–99.9) | 100.0%(94.0–100.0) |
| 316 | *sicA* |  | 99.3%(96.4–99.9) | 100.0%(94.0–100.0) |
| 317 | *spaS* |  | 99.3%(96.4–99.9) | 100.0%(94.0–100.0) |
| 318 | *spaR* |  | 99.3%(96.4–99.9) | 100.0%(94.0–100.0) |
| 319 | *spaQ* |  | 99.3%(96.4–99.9) | 100.0%(94.0–100.0) |
| 320 | *spaP* |  | 99.3%(96.4–99.9) | 100.0%(94.0–100.0) |
| 321 | *spaO* |  | 99.3%(96.4–99.9) | 100.0%(94.0–100.0) |
| 322 | *invJ* |  | 99.3%(96.4–99.9) | 100.0%(94.0–100.0) |
| 323 | *invI* |  | 99.3%(96.4–99.9) | 100.0%(94.0–100.0) |
| 324 | *invC* |  | 99.3%(96.4–99.9) | 100.0%(94.0–100.0) |
| 325 | *invB* |  | 99.3%(96.4–99.9) | 100.0%(94.0–100.0) |
| 326 | *invA* |  | 99.3%(96.4–99.9) | 100.0%(94.0–100.0) |
| 327 | *invE* |  | 99.3%(96.4–99.9) | 100.0%(94.0–100.0) |
| 328 | *invG* |  | 99.3%(96.4–99.9) | 100.0%(94.0–100.0) |
| 329 | *A225_1604* |  | 99.3%(96.4–99.9) | 100.0%(94.0–100.0) |
| 330 | *stbA* |  | 99.3%(96.4–99.9) | 100.0%(94.0–100.0) |
| 331 | *stbB* |  | 99.3%(96.4–99.9) | 100.0%(94.0–100.0) |
| 332 | *stbE* |  | 99.3%(96.4–99.9) | 100.0%(94.0–100.0) |
| 333 | *sseJ* |  | 98.7%(95.4–99.6) | 100.0%(94.0–100.0) |
| 334 | *A225_3877* |  | 98.7%(95.4–99.6) | 100.0%(94.0–100.0) |
| 335 | *bcfG* |  | 98.7%(95.4–99.6) | 98.3%(91.1–99.7) |
| 336 | *bcfF* |  | 98.7%(95.4–99.6) | 98.3%(91.1–99.7) |
| 337 | *bcfE* |  | 98.7%(95.4–99.6) | 98.3%(91.1–99.7) |
| 338 | *bcfD* |  | 98.7%(95.4–99.6) | 98.3%(91.1–99.7) |
| 339 | *bcfB* |  | 98.7%(95.4–99.6) | 98.3%(91.1–99.7) |
| 340 | *bcfA* |  | 98.7%(95.4–99.6) | 98.3%(91.1–99.7) |
| 341 | *stdB* |  | 98.0%(94.4–99.3) | 98.3%(91.1–99.7) |
| 342 | *stdC* |  | 98.0%(94.4–99.3) | 98.3%(91.1–99.7) |
| 343 | *stdA* |  | 98.0%(94.4–99.3) | 98.3%(91.1–99.7) |
| 344 | *KOX_13890* |  | 98.0%(94.4–99.3) | 98.3%(91.1–99.7) |
| 345 | *bcfH* |  | 98.0%(94.4–99.3) | 98.3%(91.1–99.7) |
| 346 | *cah* |  | 3.3%(1.4–7.4) | 1.7%(0.3–8.9) |
| 347 | *YPA_2597* |  | 1.3%(0.4–4.6) | 1.7%(0.3–8.9) |
| 348 | *pECS88_0104* |  | 1.3%(0.4–4.6) | 1.7%(0.3–8.9) |
| 349 | *pipB2* |  | 1.3%(0.4–4.6) | 0.0%(0.0–6.0) |
| 350 | *pilR* |  | 1.3%(0.4–4.6) | 0.0%(0.0–6.0) |
| 351 | *pilT* |  | 1.3%(0.4–4.6) | 0.0%(0.0–6.0) |
| 352 | *pilV* |  | 1.3%(0.4–4.6) | 0.0%(0.0–6.0) |
| 353 | *pilU* |  | 1.3%(0.4–4.6) | 0.0%(0.0–6.0) |
| 354 | *pilS* |  | 1.3%(0.4–4.6) | 0.0%(0.0–6.0) |
| 355 | *pilQ* |  | 1.3%(0.4–4.6) | 0.0%(0.0–6.0) |
| 356 | *pilO* |  | 1.3%(0.4–4.6) | 0.0%(0.0–6.0) |
| 357 | *pilM* |  | 1.3%(0.4–4.6) | 0.0%(0.0–6.0) |
| 358 | *stgA* |  | 1.3%(0.4–4.6) | 0.0%(0.0–6.0) |
| 359 | *sipC* |  | 1.3%(0.4–4.6) | 0.0%(0.0–6.0) |
| 360 | *pilK* |  | 1.3%(0.4–4.6) | 0.0%(0.0–6.0) |
| 361 | *vexE* |  | 1.3%(0.4–4.6) | 0.0%(0.0–6.0) |
| 362 | *vexD* |  | 1.3%(0.4–4.6) | 0.0%(0.0–6.0) |
| 363 | *vexC* |  | 1.3%(0.4–4.6) | 0.0%(0.0–6.0) |
| 364 | *vexB* |  | 1.3%(0.4–4.6) | 0.0%(0.0–6.0) |
| 365 | *vexA* |  | 1.3%(0.4–4.6) | 0.0%(0.0–6.0) |
| 366 | *tviE* |  | 1.3%(0.4–4.6) | 0.0%(0.0–6.0) |
| 367 | *tviD* |  | 1.3%(0.4–4.6) | 0.0%(0.0–6.0) |
| 368 | *tviC* |  | 1.3%(0.4–4.6) | 0.0%(0.0–6.0) |
| 369 | *tviB* |  | 1.3%(0.4–4.6) | 0.0%(0.0–6.0) |
| 370 | *tviA* |  | 1.3%(0.4–4.6) | 0.0%(0.0–6.0) |
| 371 | *stgD* |  | 1.3%(0.4–4.6) | 0.0%(0.0–6.0) |
| 372 | *stgB* |  | 1.3%(0.4–4.6) | 0.0%(0.0–6.0) |
| 373 | *cib* |  | 1.3%(0.4–4.6) | 0.0%(0.0–6.0) |
| 374 | *tia* |  | 1.3%(0.4–4.6) | 0.0%(0.0–6.0) |
| 375 | *sodCI* |  | 0.7%(0.1–3.6) | 0.0%(0.0–6.0) |
| 376 | *SG1048* |  | 0.7%(0.1–3.6) | 0.0%(0.0–6.0) |
| 377 | *CAA68592* |  | 0.7%(0.1–3.6) | 0.0%(0.0–6.0) |
| 378 | *SG1043* |  | 0.7%(0.1–3.6) | 0.0%(0.0–6.0) |
| 379 | *SG1042* |  | 0.7%(0.1–3.6) | 0.0%(0.0–6.0) |
| 380 | *SG1041* |  | 0.7%(0.1–3.6) | 0.0%(0.0–6.0) |
| 381 | *SG1040* |  | 0.7%(0.1–3.6) | 0.0%(0.0–6.0) |
| 382 | *SG1039* |  | 0.7%(0.1–3.6) | 0.0%(0.0–6.0) |
| 383 | *SG1038* |  | 0.7%(0.1–3.6) | 0.0%(0.0–6.0) |
| 384 | *SG1037* |  | 0.7%(0.1–3.6) | 0.0%(0.0–6.0) |
| 385 | *SG1036* |  | 0.7%(0.1–3.6) | 0.0%(0.0–6.0) |
| 386 | *SG1035* |  | 0.7%(0.1–3.6) | 0.0%(0.0–6.0) |
| 387 | *SG1033* |  | 0.7%(0.1–3.6) | 0.0%(0.0–6.0) |
| 388 | *SG1032* |  | 0.7%(0.1–3.6) | 0.0%(0.0–6.0) |
| 389 | *aatA* |  | 0.7%(0.1–3.6) | 0.0%(0.0–6.0) |
| 390 | *O3M_04325* |  | 0.7%(0.1–3.6) | 0.0%(0.0–6.0) |
| 391 | *EC55989_3339* |  | 0.7%(0.1–3.6) | 0.0%(0.0–6.0) |
| 392 | *A225_3377* |  | 0.7%(0.1–3.6) | 0.0%(0.0–6.0) |
| 393 | *hcp/tssD* |  | 0.7%(0.1–3.6) | 0.0%(0.0–6.0) |
| 394 | *KPK_3059* |  | 0.7%(0.1–3.6) | 0.0%(0.0–6.0) |
| 395 | *KOX_22410* |  | 0.7%(0.1–3.6) | 0.0%(0.0–6.0) |
| 396 | *EC55989_3319* |  | 0.7%(0.1–3.6) | 0.0%(0.0–6.0) |
| 397 | *EC55989_3315* |  | 0.7%(0.1–3.6) | 0.0%(0.0–6.0) |
| 398 | *tsh* |  | 0.7%(0.1–3.6) | 0.0%(0.0–6.0) |

*, *P*<0.05


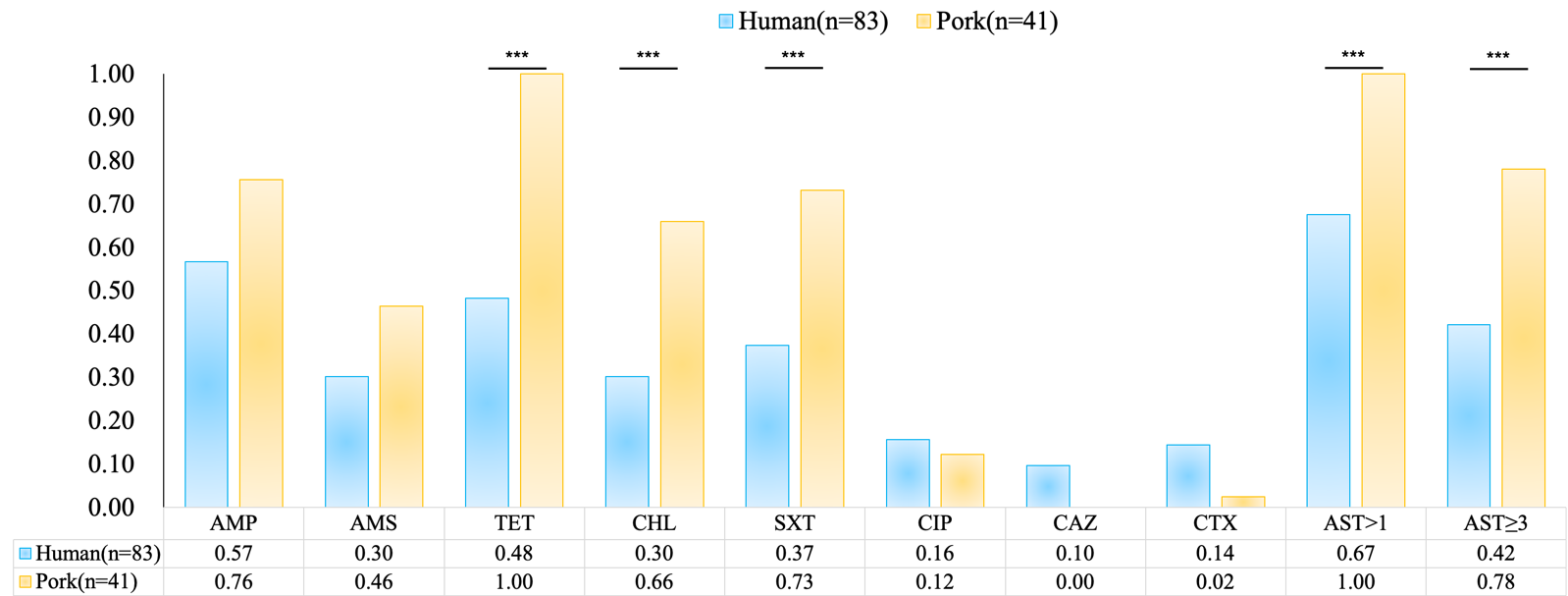


**Figure S1 Comparative analysis of antimicrobial resistance between Human- and Pork-derived *Salmonella* isolates (n=124)**.

Bar chart comparing the resistance profiles of 124 *Salmonella* isolates, including 83 human-derived and 41 pork-derived strains, which are consistent in serotype and sequence type (STs). The proportion of TET, CHL, SXT, AST>1 and AST≥3 was significantly higher in pork-derived isolates compared to human-derived isolates (*P*<0.001). **P*< 0.05; ***P*≤ 0.01; ****P*≤ 0.001


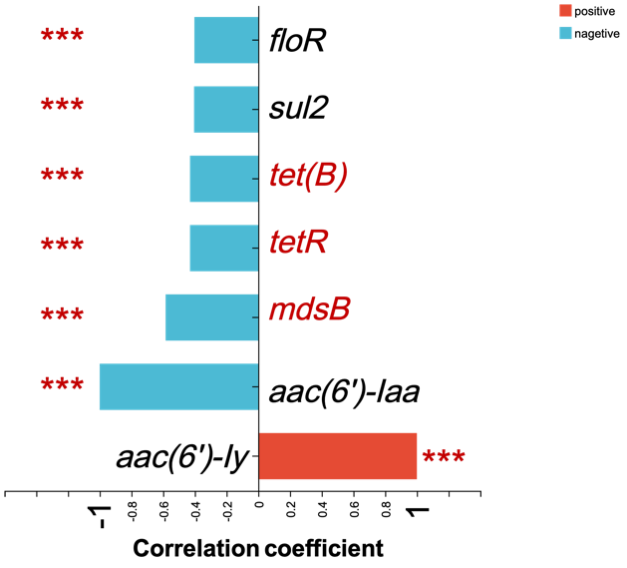


**Figure S2 Spearman correlation analysis of the *aac(6')-Iy* gene with other resistance genes**.

Significant negative correlations with tetracycline resistance genes, such as *tet(B)* and *tetR*, were observed across 124 *Salmonella* isolates with consistent serotypes and sequence types (*P* <0.001).


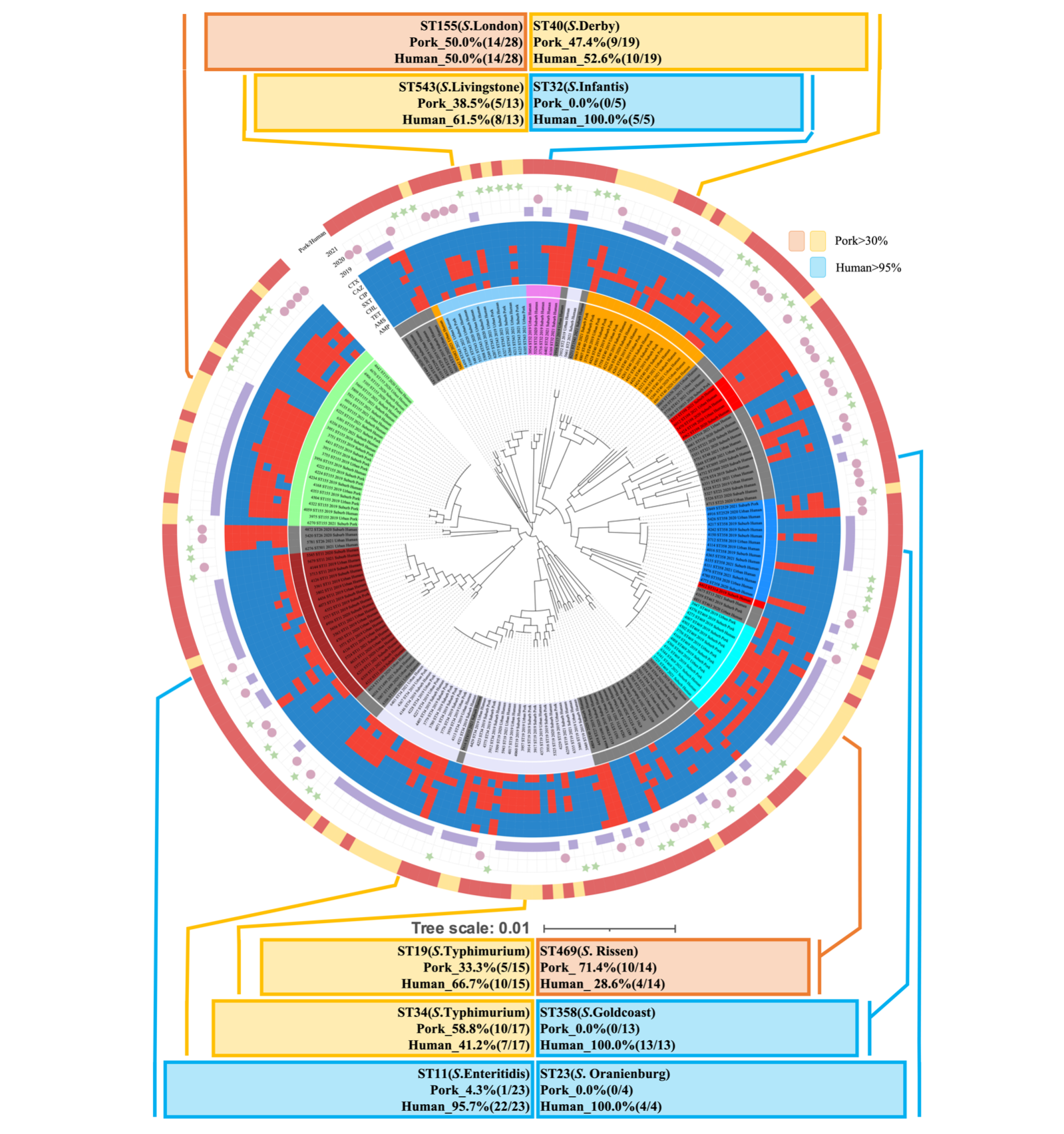


**Figure S3 The cgMLST-based phylogenetic tree of 213 *Salmonella* isolates based on the scheme developed by the INNUENDO consortium.**

The tree illustrates the genetic relationships among human-derived (n=153) and pork-derived (n=60) *Salmonella* isolates. A total of 50 sequence types (STs) were identified through in silico MLST analysis. Predominant STs, including ST11 (*S*. Enteritidis), ST155 (*S*. London), ST19 (*S*. Typhimurium), ST40 (*S*. Derby), ST543 (*S*. Livingstone), and ST469 (*S*. Rissen), were shared between human and pork sources, indicating their potential role in cross-species transmission. In contrast, ST23 (*S*. Oranienburg), ST32 (*S*. Infantis), ST198 (*S*. Kentucky), ST358 (*S*. Goldcoast), and ST1499 (*S*. Bovismorbificans) were exclusively detected in human-derived isolates in this study. A branch length of 0.01 indicates 1% allelic variation.


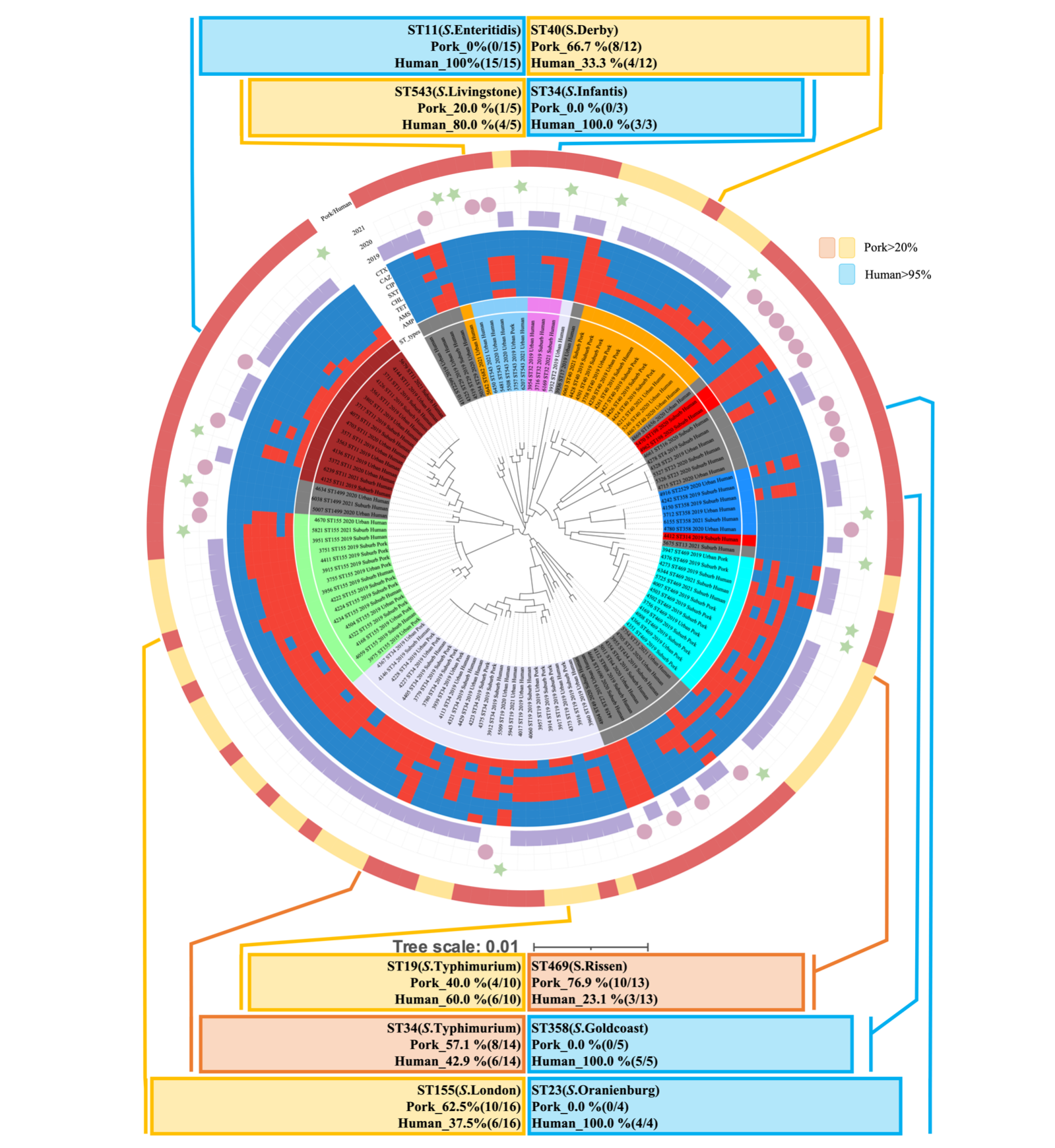


**Figure S4 The cgMLST-based phylogenetic tree of 124 *Salmonella* isolates based on the scheme developed by the INNUENDO consortium.**

The tree illustrates the genetic relationships among 124 *Salmonella* isolates with consistent serotypes and sequence types (STs), including 83 human-derived and 41 pork-derived strains. A total of 30 sequence types (STs) were identified through in silico MLST analysis. Predominant STs, including ST155 (*S*. London), ST19 (*S*. Typhimurium), ST40 (*S*. Derby), ST543 (*S*. Livingstone), and ST469 (*S*. Rissen), were shared between human and pork sources, indicating their potential role in cross-species transmission. In contrast, ST23 (*S*. Oranienburg), ST32 (*S*. Infantis), ST198 (*S*. Kentucky) and ST358 (*S*. Goldcoast) were exclusively detected in human-derived isolates in this study. A branch length of 0.01 indicates 1% allelic variation.
